# Cardiac fibroblast-derived IGFBP6 orchestrates cardiac remodeling by coupling the EGR1-MFAP4 axis

**DOI:** 10.7150/ijbs.114417

**Published:** 2025-10-01

**Authors:** Shaopeng Cheng, Yilin Wang, Kunsheng Li, Xiaoting Wu, Qianwen Zhao, Tingting Tong, Jian Shi, Yunxing Xue, Jie Yang, Dongjin Wang

**Affiliations:** 1 Department of Cardiothoracic Surgery, Nanjing Drum Tower Hospital, Affiliated Hospital of Medical School, Nanjing University, Nanjing, Jiangsu, China.; 2 Institute of Cardiothoracic Vascular Disease, Nanjing University, Nanjing, Jiangsu, China.; 3 Department of Cardiothoracic Surgery, Nanjing Drum Tower Hospital Clinical College of Nanjing University of Chinese Medicine, Nanjing, Jiangsu, China.; 4 Department of Intensive Care Unit, Nanjing Drum Tower Hospital, Affiliated Hospital of Medical School, Nanjing University, Nanjing, Jiangsu, China.; 5 Department of Infectious Disease, Nanjing Drum Tower Hospital, Affiliated Hospital of Medical School, Nanjing University, Nanjing, Jiangsu, China.; 6 Key Laboratory of Targeted Intervention of Cardiovascular Disease, Collaborative Innovation Center for Cardiovascular Disease Translational Medicine, School of Basic Medical Sciences, Nanjing Medical University, Nanjing, Jiangsu, China.

**Keywords:** cardiac fibrosis, fibroblast, IGFBP6, myofibroblast, EGR1, MFAP4

## Abstract

**Rationale:** The transition of fibroblasts into activated myofibroblasts is a pivotal driver of collagen deposition and adverse cardiac remodeling. Insulin-like growth factor-binding protein 6 (IGFBP6), a critical modulator of cellular growth and metabolism via its regulation of IGF-II activity, has been implicated in immune and fibrotic responses. However, its specific role in fibroblast-mediated cardiac remodeling, particularly in the regulation of myofibroblast transition, remains incompletely understood.

**Methods:** We analyzed IGFBP6 expression in ischemic cardiomyopathy-associated cardiac fibrosis using Gene Expression Omnibus (GEO) dataset. Serum IGFBP6 levels in patients with chronic myocardial infarction (MI) were quantified via ELISA. Cardiac fibroblast and myofibroblast-specific IGFBP6 knockout mice were generated by crossing IGFBP6 floxed (IGFBP6^f/f^) mice with tamoxifen-inducible Col1a2-Cre and Postn-MerCreMer mice. Cardiac function, tissues morphology, and molecular alterations were analyzed following MI or isoproterenol (ISO) challenge. The mechanisms underlying the regulation of fibroblast-to-myofibroblast transition (FMT) by IGFBP6 were elucidated using LC-MS/MS and RNA sequencing.

**Results:** IGFBP6 expression was significantly upregulated in cardiac fibroblasts isolated from murine fibrotic hearts and was responsive to TGF-β1 stimulation. The elevated serum IGFBP6 levels were correlated with the incidence of chronic MI. Conditional knockout of IGFBP6 in cardiac fibroblasts and myofibroblasts markedly attenuated post-MI fibrotic remodeling, ventricular dysfunction, and ISO-induced cardiac hypertrophy and fibrosis. IGFBP6 silencing abolished TGF-β1-triggered FMT. Mechanistically, TGF-β1 stimulation facilitated the translocation of IGFBP6 in cardiac fibroblasts, where its N-terminal domain directly interacted with early growth regulator 1 (EGR1). This interaction enhanced EGR1 binding to the promoter of microfibril-associated protein 4 (MFAP4), a pro-fibrotic mediator. Overexpression of MFAP4 significantly reversed the protective effects by IGFBP6 knockout in cardiac fibroblast transition and adverse remodeling post-MI.

**Conclusion:** Our study identifies fibroblast-derived IGFBP6 as a novel regulator of cardiac fibrosis through the EGR1-MFAP4 signaling axis, driving myofibroblasts differentiation and adverse remodeling. Targeting this pathway may offer therapeutic potential for cardiac remodeling disorders.

## Introduction

Sustained physiological or pathological cardiac stress results in excessive production of extracellular matrix (ECM) components, leading to cardiac fibrosis 1. Cardiac fibrosis disturbs the architecture of the myocardium, exacerbates cardiac dysfunction, and significantly influences the clinical progression and outcome in patients with heart failure 2-4. It is reported that fibrosis is independently associated with increased cardiovascular and all-cause mortality 5. Despite its role in heart failure, there are currently no effective therapeutic strategies that specifically target cardiac fibrosis.

Cardiac fibroblasts (CFs) constitute most of the non-myocyte cell population in the heart and are the principal effector cells causing fibrosis 5-7. Following injury, CFs transdifferentiate into hyperactive myofibroblasts under biomechanical (e.g., TGF-β1) cues, driving pathological ECM deposition and inflammatory crosstalk via cytokine networks 8. While the initial fibrotic response to myocardial injury is considered to be an adaptive and protective mechanism, persistent fibrosis leads to irreversible structural changes in the ventricles, ultimately impairing cardiac function 9. Additionally, CFs play a pivotal role in modulating immune cell recruitment through the release of cytokines 10. Therefore, elucidating the molecular interactions between CFs and myofibroblasts in fibrotic myocardium is crucial for identifying novel therapeutic targets to mitigate adverse cardiac remodeling.

Insulin-like growth factor-binding protein 6 (IGFBP6) is a secreted protein that binds insulin-like growth factors (IGFs) and is a member of the IGFBP family (IGFBP1 to IGFBP7). This family comprises proteins capable of binding to and modulating IGF activity 11. Unlike other IGFBPs, IGFBP6 exhibits a 50-fold higher affinity for IGF-II compared with IGF-I 12. Emerging evidence suggest that IGFBP6 may exert biological functions independently of IGF-II, including the regulation of cell proliferation, apoptosis, migration and angiogenesis 13-15. IGFBP6 is highly expressed in hepatic stellate cells and is strongly correlated with hepatic fibrosis, steatosis severity, and a non-alcoholic fatty liver disease (NAFLD) activity score 16. Additionally, IGFBP6 has shown to promote the activation of the Toll-like receptor 4 (TLR4) signaling pathway and aggravate myelofibrosis by regulating the Sonic Hedgehog (SHH) signaling cascade 17. During tissue damage repair, IGFBP6 also plays a role in immune-inflammatory responses by recruiting and activating various immune cell types, thereby influencing the nature of inflammation 18-20. These findings underscore the pivotal role of IGFBP6 in promoting fibrosis and inflammatory response. However, the specific mechanism by which IGFBP6 contributes to cardiac fibrosis, particularly in the context of myofibroblast transition, remains poorly understood.

In the present study, we identify IGFBP6 as a mechanosensitive mediator predominantly upregulated in cardiac fibroblasts from murine fibrotic myocardium. This matricellular protein demonstrates TGF-β1-responsive expression patterns and critically regulates fibroblast-to-myofibroblast transition. Genetic ablation of IGFBP6 specifically in CFs and myofibroblasts significantly attenuates pathological ventricular remodeling and prevents cardiac dysfunction with MI or ISO challenge. Mechanistically, TGF-β1 stimulation initiates nuclear translocation of IGFBP6 via its nuclear localization signal (NLS), followed by physical interaction between the N-terminal domain of IGFBP6 and EGR1. This molecular complex subsequently facilitates EGR1-mediated transcriptional activation of microfibril-associated protein 4 (MFAP4) through direct promoter binding. Our findings establish cardiac fibroblast-derived IGFBP6 as a novel amplifier of fibrotic signaling cascades and propose a targetable axis for therapeutic intervention in ischemic cardiomyopathy.

## Materials and Methods

For detailed methods and study materials, please refer to the Supplementary Materials.

### Human samples

Human serum samples from heathy individuals were obtained from the health examination center (n=24), and those from patients with myocardial infarction (MI) without surgery or percutaneous coronary intervention (PCI) treatment from the department of Cardiothoracic Surgery (n=32). The serum samples were used for ELISA experiments. Written informed consent was obtained from the patients or the families of donors included in this research. The study was approved by the Institutional Ethics Committee of Nanjing Drum Tower Hospital (approval number: NO. SC2022711 and NO. 2024-482-02) and adhered to the ethical guidelines of the 1975 Declaration of Helsinki. The clinical characteristics of the patients are provided in **Supplementary Table S1**.

### Animals

All animal experiments were conducted in compliance with the guidelines for the Care and Use of Laboratory Animals, as published by the US National Institutes of Health (NIH Publication, 8th Edition, 2011) and were approved by the Nanjing University Drum Tower Hospital Committee of Animal Care (Permit No. IACUC2308005). The mice were housed in a specific pathogen-free (SPF) environment with a 12-hour light/dark cycles and were provided *ad libitum* access to food and water. Male C57BL/6JGpt mice (Strain NO. N000013) were purchased from GemPharmatech (Nanjing, China). Male C57BL/6JGpt mice, aged 6-8 weeks, were used for developing the MI model, transverse aortic constriction (TAC) model, or isoproterenol (ISO) model. Male C57BL/6JGpt mice, aged 5-6 weeks, were used for the isolation and culture of CFs. Conditional transgenic mice targeting the IGFBP6 gene (IGFBP6^f/f^) were generated by GemPharmatech (Nanjing, China) in the C57BL/6J background. The Col1a2-Cre/ERT mice and the Postn-MerCreMer mice were purchased from Cyagen Biosciences Inc. (Suzhou, China). The IGFBP6^f/f^ mice were bred with Col1a2-Cre/ERT mice and Postn-MerCreMer mice to generate fibroblast-specific and myofibroblast-specific IGFBP6 knockout in the adult mice. The mice were intraperitoneally treated with tamoxifen (30 mg/kg) dissolved in corn oil for 5 consecutive days. For the surgery, ISO injection, and virus injection, all mice were anesthetized with 1.5% isoflurane. For tissue harvesting, animals were euthanized using CO_2_ for 1 min-3 min in their home cages with a flow rate of 10%-20% of the cage volume per minutes. Detailed information is provided in the described in **Supplementary material**.

### Construction of recombinant adeno-associated virus and injection

Recombinant adeno-associated virus 9 (rAAV9) with the specific promoter Col1a2 supplied by Zebrafish Biotech Co., Ltd (Nanjing, China) was used to manipulate MFAP4 expression in mice CFs. The sequence of scramble as follows: TTCTCCGAACCGTGTCACGT. Six-week-old mice were intravenously injected with rAAV9 (3×10^11^ vg/mouse) through the tail vein for 2 weeks before MI surgery. The efficiency of viral transfection was assessed by measuring MFAP4 expression through western blotting and immunofluorescence analyses.

### Myocardial infarction models

MI was induced by ligation of the left anterior descending (LAD) coronary artery 21. Briefly, male C57BL/6JGpt mice, IGFBP6^f/f^ mice or cardiac fibroblast (IGFBP6^CF-KO^) and myofibroblast specific knockout (IGFBP6^MF-KO^) IGFBP6 mice aged 8 weeks were anesthetized with 1.5% isoflurane and maintained positive airway pressure using a rodent ventilator. The LAD coronary artery was permanently ligated with a 6-0 prolene suture. Meanwhile, a similar procedure was performed in sham-operated mice, except for LAD coronary artery ligation. The mice were sacrificed, and their heart samples were obtained for corresponding analyses at 14 days after the surgery. The border zone of the MI-operated heart was defined as the immediate neighboring regions around the infarction as seen under the stereomicroscope and the posterior wall of the left ventricle. The border and remote zones were stereologically identified prior to tissue collection and analysis.

### Transverse aortic constriction (TAC) model

A mouse model of cardiac fibrosis was established using transverse aortic constriction (TAC). Male C57BL/6JGpt mice, aged 8 weeks, were anesthetized with 1.5% isoflurane. The left thoracic cavity was opened to expose the aorta; a 27-gauge needle was placed parallel to the transverse aorta, and the aorta was ligated using 5-0 silk sutures between the first and second branches of the aortic arch. After ligation, the needle was removed, leaving the aorta constricted to the diameter of the needle. The same procedure was performed in the sham control group, except for the ligation step. The heart samples were harvested at 4 weeks after the surgical operation for qRT-PCR and western blotting analyses.

### ISO injection models

For the heart failure models, 8-week-old male C57BL/6JGpt mice, IGFBP6^f/f^ mice, IGFBP6^CF-KO^ and IGFBP6^MF-KO^ mice were administered isoproterenol (ISO, S2566, purchased from Selleck) via subcutaneous injection (10 mg/kg/day) for a duration of 2 weeks. After the models were successfully established, the mice were sacrificed, and their heart samples were collected for subsequent analyses.

### Echocardiography analysis

Cardia function was assessed using two-dimensional guided M-mode transthoracic echocardiography (VisualSonics VeVo 2100 Imaging System, Toronto, Canada), with a 30-MHz linear transducer. Cardiac function was measured both before modeling and prior to tissue collection. All the measurements were performed by a single echocardiographer who was blinded to the experimental conditions. Detailed information is provided in the **Supplementary Materials**.

### Enzyme-linked immunosorbent assay (ELISA)

Serum IGFBP6 levels in the murine and human samples were measured using a Mouse IGFBP6 ELISA Kit (EMIGFBP6**,** ThermoFisher Scientific) and Human IGFBP6 ELISA Kit (EHIGFBP6**,** ThermoFisher Scientific), respectively. ELISAs were carried out following the manufacturer protocols. Briefly, the samples were added to wells coated with antibodies and incubated at 25 °C for 2 h. The solution was discarded, and the plate was washed four times. The detection antibody bound to the fixed target protein was added to each well and incubated at 25°C for 2 h. Excess detection antibody was washed off, followed by the addition of streptavidin-HRP and incubation at 25 °C in the dark for 30 min. The plates were washed four times again and incubated for another 30 min at 25 °C in the dark after adding the substrate. The stop solution was then added, and the absorbance was measured at 450 nm after thoroughly mixing the contents.

### Cell culture

Adult mouse cardiac fibroblasts and cardiomyocytes were isolated from 6-week-old mice as previously described 22. HEK293T cells (CRL-3216, ATCC) were cultured in Dulbecco's Modified Eagle Medium (DMEM) supplemented with 10% Fetal Bovine Serum (FBS), penicillin and streptomycin. Detailed information is provided in the **Supplementary Materials**.

### Plasmids transient transfection

The HA-tagged full-length IGFBP6 (LV-3×HA-IGFBP6-WT, 1-240aa), HA-IGFBP6-N-terminal IGFBP6-superfamily domain (LV-3×HA-IGFBP6-1-107aa), and HA-IGFBP6-C-terminal thyroglobulin-1 domains (LV-3×HA-IGFBP6-108-240aa) and Flag-tagged EGR1 plasmids were constructed by Miao Ling Plasmid Sharing Platform (Wuhan, China). For transient transfection, HEK293T (CRL-3216, ATCC) cells were transfected with the indicated plasmids using Lipofectamine 3000 (#1742691, Life) according to manufacturer's recommendations.

### Lentivirus transfection

Lentiviruses carrying HA-tagged WT full-length IGFBP6 (HA-IGFBP6-WT) and HA-tagged ΔNLS IGFBP6 (HA-IGFBP6-ΔNLS) were constructed according to the instructions. The primers were as follows:

IGFBP6-ΔNLS-mut-Forward: aagcagcagtgtcgttcctcgcaggggaatcgccaaGGCCCCTGCTGGTGTGTG

IGFBP6-ΔNLS-mut-Reverse: gaacgacactgctgcttttgGTAGAAGCCTCTGAGGTCACAGTTT

Lentiviruses carrying the HA-EGFP-Vector were also constructed. After 24 hours of plating, cardiac fibroblasts were infected with lentiviruses diluted in DMEM medium (MOI = 100), and 5 μg/mL polybrene was added to enhance transfection efficacy. The medium was replaced with virus-free medium 24 hours after infection, and then cells were cultured for an additional 48 h prior to experiments.

### siRNA transfection Interference

Small interfering RNA (siRNA) targeting mouse IGFBP6 and negative control siRNA were designed, synthesized and validated by Zebrafish Biotech Co.,Ltd. The IGFBP6 siRNA sequences were as follows: Forward 5'-GCUGCUCAUGCUGCUAAUGTT-3', Reverse: 5'-CAUUAGCAGCAUGAGCAGCTT-3'. The siRNA was transfected into cells using Lipofectamine® RNAiMAX Reagent (Invitrogen, 13778075) according to the manufacturer's instructions. At the culmination of a 48-hour period following siRNA transfection, cells were harvested and used for further experiments.

### Quantitative real-time PCR

Total RNA was extracted using the TRIzol reagent (#TKR-9109, Takara, Japan) and reverse transcribed into complementary DNA using HiFScript All-in-one RT Master Mix (Cat. No. CW3371, Cowin Biotech Co, Ltd, Jiangsu, China) according to the manufacturer's instructions. qRT-PCR was performed using SuperStar Universal SYBR Master Mix (Cat. No. CW3360, Cowin Biotech Co, Ltd, Jiangsu, China) on a CFX96 real-time PCR System (Bio-Rad Laboratories, Inc., CA, USA). The mRNA levels were determined from the mean threshold cycle (Ct) values and normalized to the Ct of the internal control, unless stated otherwise. Fold changes were calculated using the 2^-ΔΔCt^ method, and the results were presented as mean ± standard deviation (SD) relative to the control. The primer sequences used in this study are provided in **Supplementary Table S2**.

### Nuclear and cytosolic fractionation

The separation of the nucleus and cytoplasm was conducted using a nuclear extraction kit (Beyotime Biotechnology, product code P0027) in accordance with the manufacturer's protocol. The isolated components were subsequently analyzed via SDS-PAGE followed by western blotting.

### Western blotting

Protein extraction was performed on both myocardium and cultured cells respectively. Immunoblotting was performed as previously described 23, and detailed information is provided in the Supplementary Materials.

### Co-immunoprecipitation

Tissues or cell lysates were extracted using a lysis buffer (40 mM Hepes, pH 7.4, 2 mM EDTA, 10 mM pyrophosphate, 10 mM glycerophosphate, 0.5% TritonX-100) supplemented with protease inhibitor cocktail (#78438, Thermo Fisher Scientific). The supernatants were collected after centrifugation at 12,000 rpm for 10 min. The cell lysates were incubated overnight at 4°C with antibodies against IgG, IGFBP6, EGR1, HA-tag, or Flag-tag, and followed by precipitation with recombinant Protein A/G covalently bound to DynaGreen magnetic beads (Cat: 80106G, Thermo Fisher Scientific) for 4 hours at 4°C. After washing, the immunoprecipitated complexes were immediately identified by SDS-PAGE and western blotting analyses.

### Luciferase assays

DNA transfection and luciferase assays were carried out following the Dual-Luciferase reporter assay system. HEK293T cells were cultured in a 24-well plate one day before transfection with 1×10^5^ cells per well. WT and mutant MFAP4 promoters were cloned into pGL6 luciferase reporter vectors. The recombinant pGL6 dual luciferase reporter vectors were co-transfected into the HEK293T cells with or without the EGR1-encoding plasmid. Subsequently, the fluorescein-labeled reporter gene activity was detected using a Dual-Luciferase Reporter Assay System kit (#E1910, Promega), according to the manufacturer's instructions. Renilla luciferase activity was normalized based on firefly luciferase activity.

### Chromatin immunoprecipitation (ChIP) assays and sequencing

ChIP-PCR was performed on mouse cardiac fibroblasts using CHIP Assay Kit (#P2078, Beyotime Biotechnology). Chromatin was sheared into 100-200 bp DNA fragments by ultrasound using a Biorupter. Detailed information is provided in the Supplementary Materials.

### RNA sequencing (RNA-seq) and data analysis

Library construction and sequencing were performed by Sinotech Genomics Co., Ltd. (Shanghai, China). Total RNA was extracted using the TRIzol reagent (Invitrogen, USA) according to the manufacturer's protocol. RNA purity and concentration were assessed using the NanoDrop 2000 spectrophotometer (Thermo Scientific, USA). RNA integrity was evaluated using the Agilent 2100 Bioanalyzer (Agilent Technologies, USA). Libraries were then constructed using the TruSeq Stranded mRNA LT Sample Prep Kit (Illumina, USA) according to the manufacturer's instructions and sequenced on an Illumina HiSeq X Ten platform, generating 150 bp paired end reads. Raw data (FASTQ format) were first processed using Trimmomatic, and the low-quality reads were removed to obtain the clean reads. The clean reads were mapped to the mouse genome (Mus_musculus. GRCm38.99) using HISAT2. The FPKM (fragments per kilobase per million mapped fragments) for each gene was calculated using Cufflinks, and read counts were obtained using HTSeq-count. Differential expression analysis was performed using the DESeq (2012) R package. Differentially expressed genes (DEGs) for each pairwise comparison were selected based on a p value < 0.05 and a fold change > 1.5. Hierarchical clustering analysis of DEGs was performed to illustrate the expression pattern of genes across different groups and samples. Gene ontology (GO) enrichment and Kyoto Encyclopedia of Genes and Genomes (KEGG) pathway enrichment analyses of DEGs were performed on R based on the hypergeometric distribution.

### Statistical analysis

Data are presented as the mean ± SD and represent the aggregate results of independent experimental replicates. For comparisons between two groups, the unpaired two-tailed Student's t-test was applied when data passed normality, as well as equal variance tests; otherwise, the Mann-Whitney U test was used. Comparisons between multiple groups were evaluated using one-way or two-way analysis of variance (ANOVA) followed by Bonferroni's multiple comparisons test where appropriate. Statistical significance was set at p < 0.05; ns denoted no significant difference. All the statistical analyses were conducted using the GraphPad Prism software (version 9.0).

## Results

### IGFBP6 is upregulated in cardiac fibroblasts during murine and human cardiac remodeling

**Figure 1:**
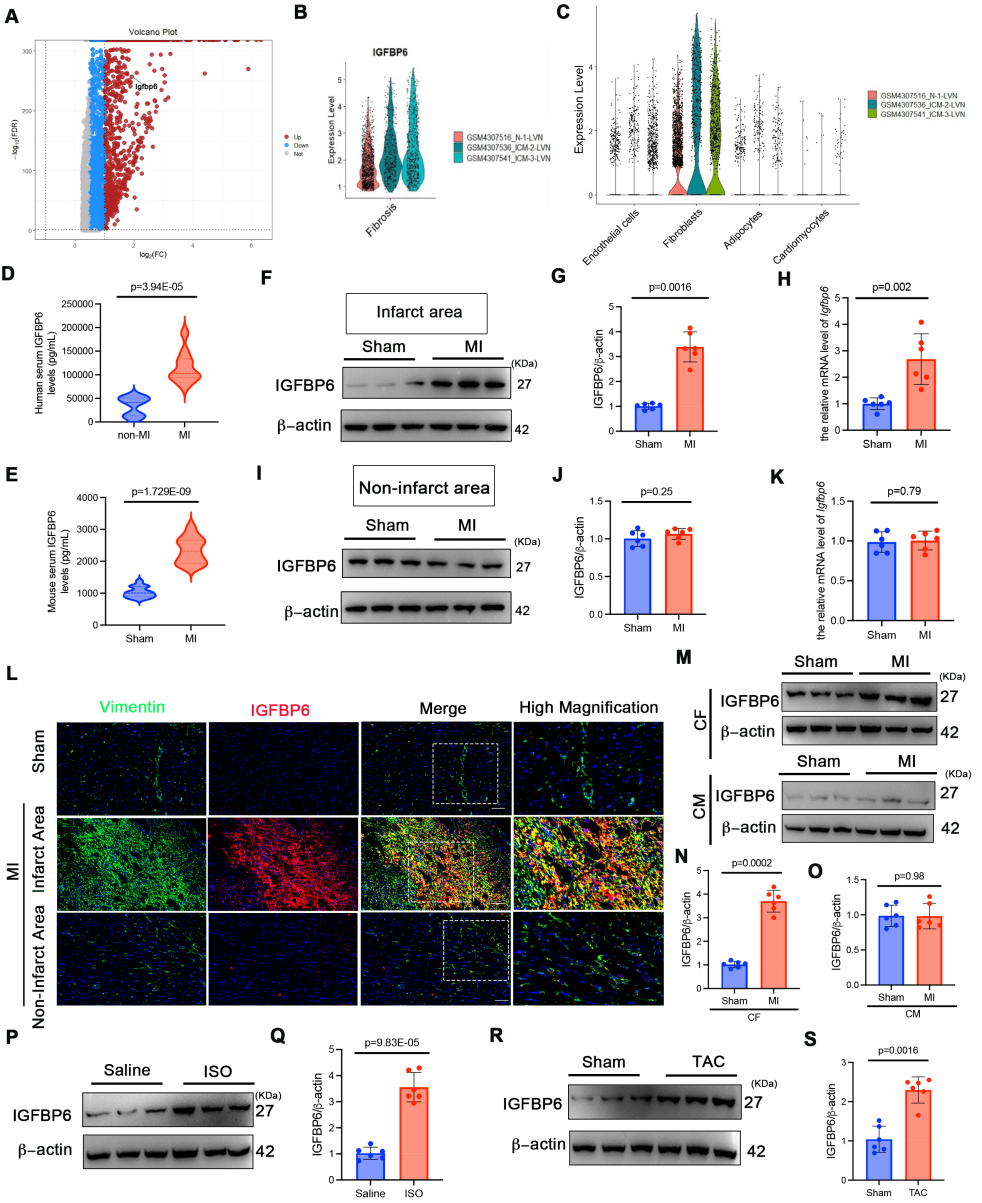
** IGFBP6 expression is increased in ischemia myocardium and fibrotic myocardium. A:** RNA sequencing analysis of IGFBP6 in ischemic cardiomyopathy (ICM) database (GSE145154). Expression profile of proteins showing up (red) and down (blue) genes. **B:** Quantitative of IGFBP6 in ICM samples. **C:** IGFBP6 expression in different cell types during normal and ICM samples. **D:** Serum IGFBP6 levels of patients with chronic MI accompanied by ventricular aneurysm versus healthy subjects were measured by ELISA (MI, n=32; healthy, n=24). **E:** C57BL/6J mice were subjected to sham or MI surgery for 2 weeks. Serum IGFBP6 levels of MI mice and sham subjects were measured by ELISA (MI, n=20; Sham, n=20). **F-K:** Western blotting and qRT-PCR were performed on the heart lysates from the infarct zones and non-infarct zones at 2 weeks post-MI to analyze the expression of IGFBP6, n=6 mice per group. **L:** Immunofluorescence co-staining for vimentin (green) with IGFBP6 (red) and DAPI (blue) in the heart post-MI. Scale bars:20 µm, n=6 mice per group. **M-O:** IGFBP6 protein expression was measured in cardiomyocytes and fibroblasts isolated from the sham or MI heart on 14 d after the surgery. n=6 mice per group. **P-Q:** WT mice were injected ISO (10 mg/kg/d) for 14 days to induce cardiac fibrosis. Western blotting was used to detect the expression of IGFBP6, n=6 mice per group. **R-S:** WT mice were subjected TAC or Sham surgery for 4 weeks. Western blotting analysis and quantification of IGFBP6 were shown. n=6 mice per group. Data are expressed as mean ± SD. Two tailed Student's *t-*test were used for analysis.

To investigate the role of IGFBP6 in cardiac fibrosis, we reanalyzed the RNA-seq dataset from patients with ischemic cardiomyopathies (ICM: GSE145154). As shown in **Figure 1A-B**, the IGFBP6 levels were elevated in ICM and myocardial fibrotic tissues compared with controls. Furthermore, IGFBP6 expression was mainly localized to fibroblasts (**Figure 1C**). Consistent with the analysis of GEO datasets, ELISA quantification revealed elevated serum IGFBP6 levels in patients with chronic MI accompanied by ventricular aneurysm, compared with those in healthy controls (**Figure 1D**, clinical characteristics in **Supplementary Table S1)**. To validate these findings experimentally, we induced cardiac remodeling in mice through MI surgery. Serum IGFBP6 levels were markedly increased 14 days post-surgery in MI-operated mice compared with sham controls (**Figure 1E**). IGFBP6 expression was increased mainly in the peri-infarcted heart after MI at different time points (**Figure S1A-C**). We then analyzed the expression of IGFBP6 in different zones. In the infarct zone, MI induced the dramatical elevation in IGFBP6 protein levels; no detectable changes in IGFBP6 expression were observed in the non-infarct area (**Figure 1F-K**). Immunofluorescent co-staining further revealed the increase of IGFBP6 and vimentin double-positive cells in the infarct zone on 14 days post-MI, but not in the non-infarct zone (**Figure 1L**). We next isolated CFs and cardiomyocytes from sham and MI hearts at day 14 post-MI. While IGFBP6 was markedly increased in the fibroblasts from MI hearts, its expression in cardiomyocytes remained unchanged (**Figure 1M-O**). In addition, the fibrotic myocardium obtained from ISO-induced mice expressed higher IGFBP6 mRNA and proteins than the hearts harvested from saline controls (**Figure 1P-Q, Fig S1D**). TAC could also induce elevated IGFBP6 expression levels in the fibrotic myocardium (**Figure 1R-S, Figure S1E**). Collectively, these data establish IGFBP6 as a conserved marker of activated fibroblasts across multiple cardiac injury models.

### IGFBP6 contributes to TGF-β1-induced myofibroblast transformation *in vitro*

**Figure 2:**
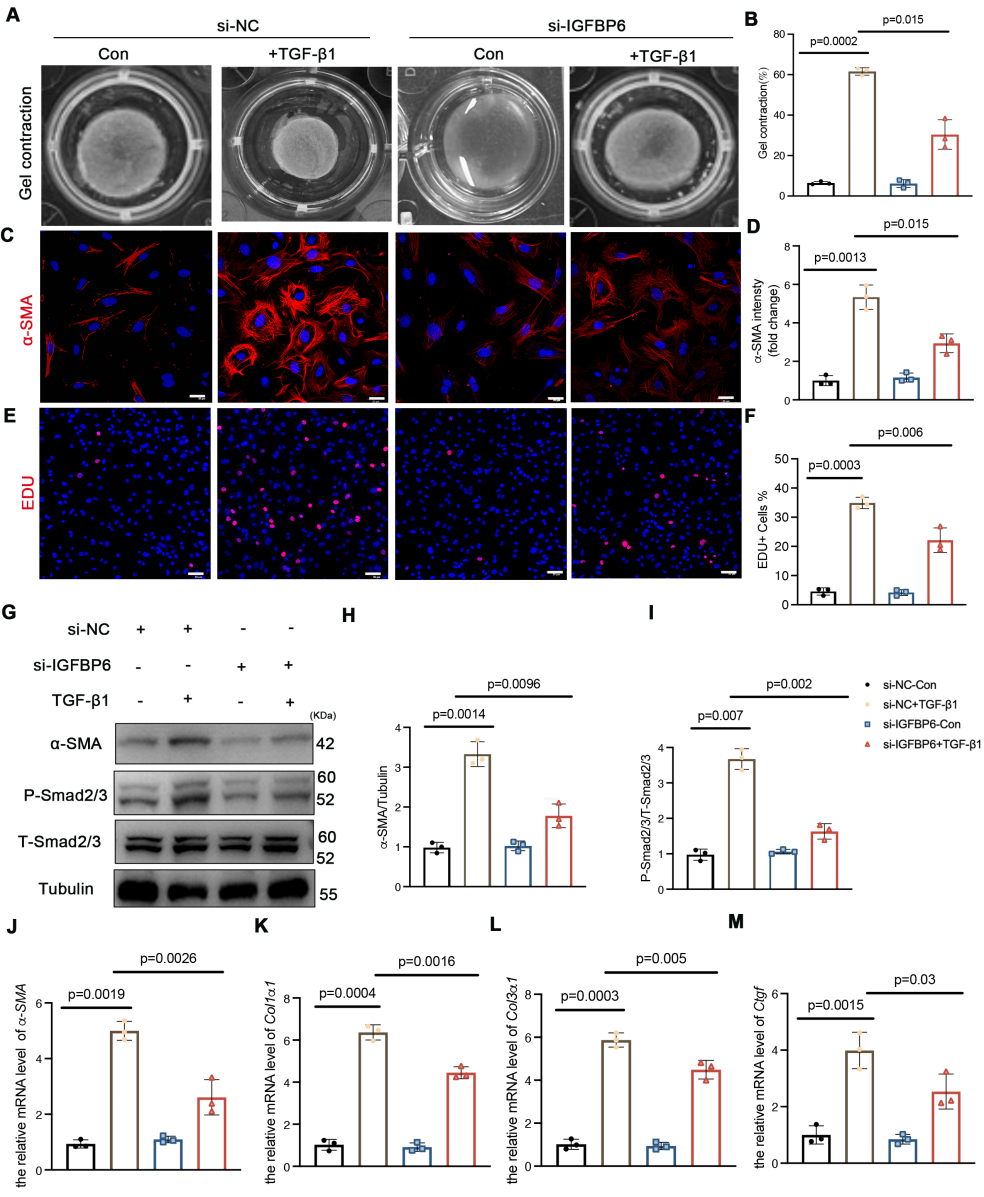
** Silencing of IGFBP6 expression blocks TGF-β1-induced myofibroblast transition.** Primary cardiac fibroblasts were isolated from the 6-week-old male C57BL/6J mice. Fibroblasts were transfected with small interfering RNA targeting IGFBP6 (si-IGFBP6) or negative control (si-NC) followed by exposure to vehicle or TGF-β1 (10 ng/mL) for 48 h. **A-B:** Collagen contractility with representative collagen gels showing contraction 48 h after the gel release, with percent collagen gel contraction quantified over 48 h period. **C-D:** Immunofluorescence staining was performed to detect the expression of α-SMA (red). Nuclei was stained with DAPI (blue). Scale bars: 20 µm. **E-F:** Representative images and quantification of the proliferation of cardiac fibroblasts with the Edu (red) staining. Scale bars: 50 µm. **G-I:** Immunoblots and quantification of α-SMA, P-Smad2/3 and T-Smad2/3. Tubulin was used as reference. **J-M:** mRNA expression levels of α-SMA, Col1α1, Col3α1, Ctgf were detected by real-time quantification PCR. n=3 independent biological. Data are expressed as mean ± SD. Two-way ANOVA followed by Tukey's multiple comparisons test.

CFs serve as pivotal effector cells in mediating both reparative and pathological fibrotic processes following MI, primarily through fibroblast-to-myofibroblast transition. To investigate the potential involvement of IGFBP6 in fibroblast-to-myofibroblast transition, we first examined its expression dynamics in TGF-β1-stimulated CFs. Western blotting and qPCR analysis revealed time-dependent upregulation of IGFBP6 protein and mRNA levels upon treatment with 10 ng/mL TGF-β1 (**Figure S2A-C**). To functionally characterize the role of IGFBP6, we transfected CFs with si-RNA (si-IGFBP6) and negative control siRNA (si-NC). The transfection efficiency was validated by significant reduction in IGFBP6 protein expression in si-IGFBP6-treated cells compared with si-NC controls (**Figure S2D-E**). The contraction of collagen gels assay revealed that silencing of IGFBP6 reversed the contractile capacity of the CFs with the TGF-β1 stimulation (**Figure 2A-B**). Both α-SMA fluorescence staining and Edu staining demonstrated that silencing of IGFBP6 attenuated TGF-β1-induced myofibroblast transition (**Figure 2C-F**). Immunoblotting showed that silencing of IGFBP6 downregulated α-SMA and P-Smad2/3 protein expression with the TGF-β1 stimulation in CFs (**Figure 2G-I**). The mRNA expression levels of myofibroblast marker genes (*α-SMA, Col1α1, Col3α1, and Ctgf*) were also decreased in IGFBP6 knockdown CFs upon TGF-β1 exposure (**Figure 2J-M**). These findings indicates that IGFBP6 is involved in the transformation of myofibroblasts and that silencing of IGFBP6 is sufficient to suppress this phenomenon.

### Cardiac fibroblast-specific knockout of IGFBP6 attenuates cardiac fibrosis

**Figure 3:**
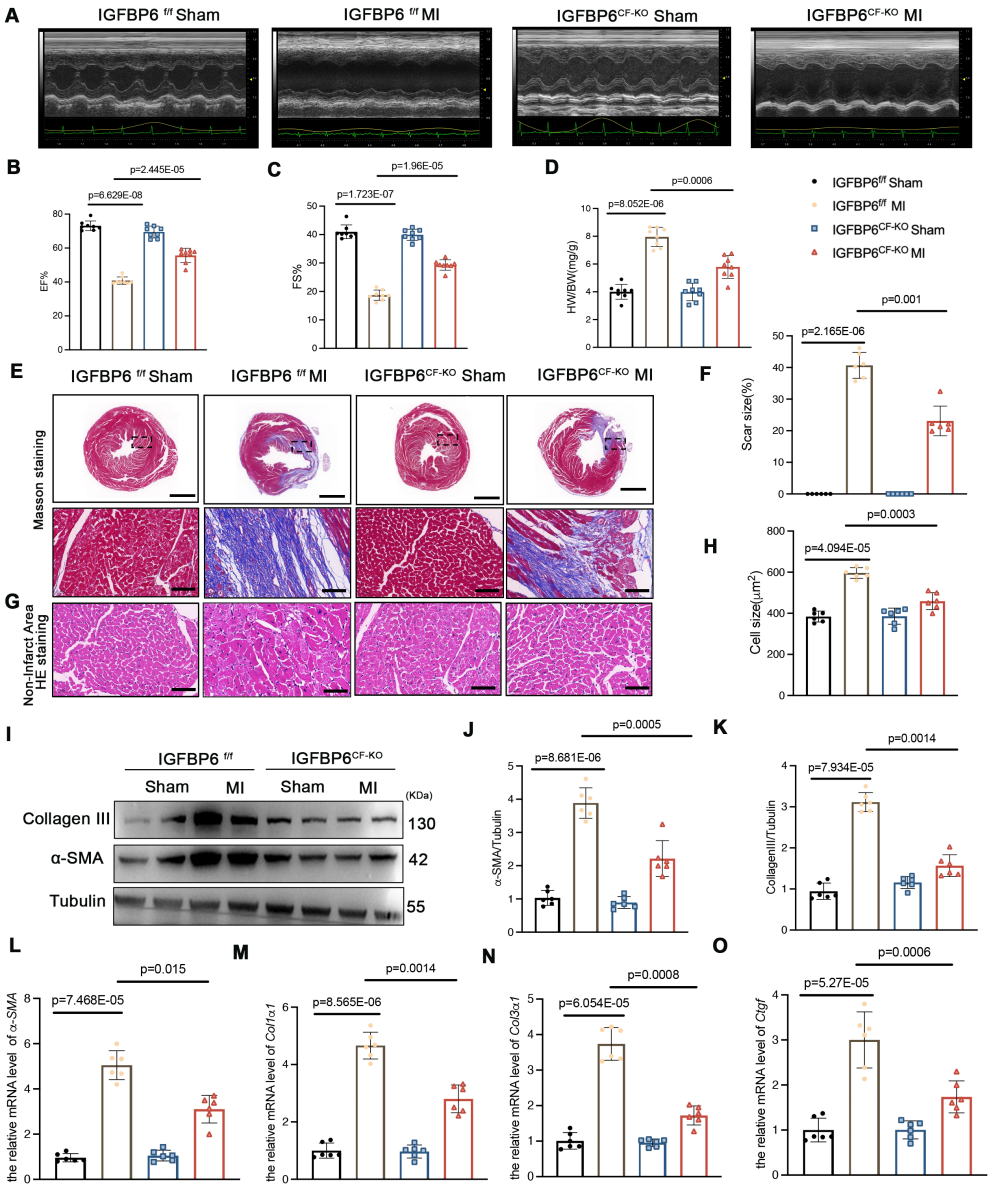
** Cardiac fibroblast-specific IGFBP6 deficiency attenuates adverse remodeling and cardiac dysfunction after MI.** IGFBP6^f/f^ and IGFBP6^CF-KO^ mice were injected tamoxifen for 5 continuous days and then subjected to MI or Sham surgery. Cardiac function indices were measured by echocardiography. **A:** Representative M-mode echocardiographic images of IGFBP6^f/f^ and IGFBP6^CF-KO^ mice. **B-C:** Quantification of cardiac EF (ejection fraction) and FS (fractional shortening). n=8 mice per group.** D:** Quantification of HW/BW (heart weight/body weight) between IGFBP6^f/f^ and IGFBP6^CF-KO^ mice. n=8 mice per group. **E-F:** Representative images and quantification of heart sections stained with Masson trichrome staining at 2 weeks after MI surgery in IGFBP6^f/f^ and IGFBP6^CF-KO^ mice. Scar circumference was measured and expressed as a percentage of total circumstance of left ventricle. n=6 mice per group. Scale bars: 500 µm and 50 µm. **G-H:** Representative images and quantification of cell size in left ventricular border zone. n=6 mice per group. Scale bars: 50µm. **I-K:** Western blot and analysis of α-SMA and Collagen III in the infarct zone at 2 weeks post-MI. n=6 mice per group. **L-O:** mRNA expression levels of α-SMA, Col1α1, Col3α1, Ctgf were detected by real-time quantification PCR. n=6 mice per group. Data are expressed as mean ± SD. Two-way ANOVA followed by Tukey's multiple comparisons test was used for analysis.

To delineate the function of IGFBP6 in CFs during cardiac fibrosis, we generated cardiac fibroblast-specific IGFBP6 knockout mice (IGFBP6^CF-KO^) by crossing IGFBP6-floxed (IGFBP6^f/f^) mice with Col1a2-cre/ERT mice (**Figure S3A**). This strategy enabled tamoxifen (TAM)-inducible deletion of IGFBP6 specifically in CFs. Genotyping confirmed successful recombination in IGFBP6^CF-KO^ mice, and five consecutive days of TAM administration effectively reduced IGFBP6 expression in the CFs isolated from IGFBP6^CF-KO^ mice compared with those isolated from IGFBP6^f/f^ controls (**Figure S3B-F**). Cardiac functions of IGFBP6^f/f^ and IGFBP6^CF-KO^ mice were not significantly different at baseline (**Supplementary Table S3**). All the mice were then subjected to MI surgery or a sham operation. Initial assessment at 3 days post-MI revealed comparable infarct sizes in the IGFBP6^f/f^ and IGFBP6^CF-KO^ mice (**Figure S3G-H**). However, echocardiography analysis at 2 weeks demonstrated that IGFBP6 deficiency in the CFs significantly ameliorated MI-induced cardiac dysfunction, as evidenced by improved ejection fraction (EF) and fractional shortening (FS) (**Figure 3A-C, Supplementary Table S4**). IGFBP6 deficiency in the CFs significantly decreased heart weight/body weight (HW/BW) post-MI when compared with IGFBP6^f/f^ mice (**Figure 3D**). Histological analysis further revealed that IGFBP6^CF-KO^ mice exhibited reduced infarct size and attenuated cardiomyocyte hypertrophy in non-ischemic regions compared with IGFBP6^f/f^ mice (**Figure 3E-H**). The *α*-SMA and collagen III protein levels were significantly decreased in the IGFBP6^CF-KO^ mice remodeling myocardium (**Figure 3I-K**). Consistent with these findings, the mRNA levels of *α-SMA, Col1α1, Col3α1* and* Ctgf* were also lower in IGFBP6^CF-KO^ mice than in IGFBP6^f/f^ mice post-MI (**Figure 3L-O**).

**Figure 4:**
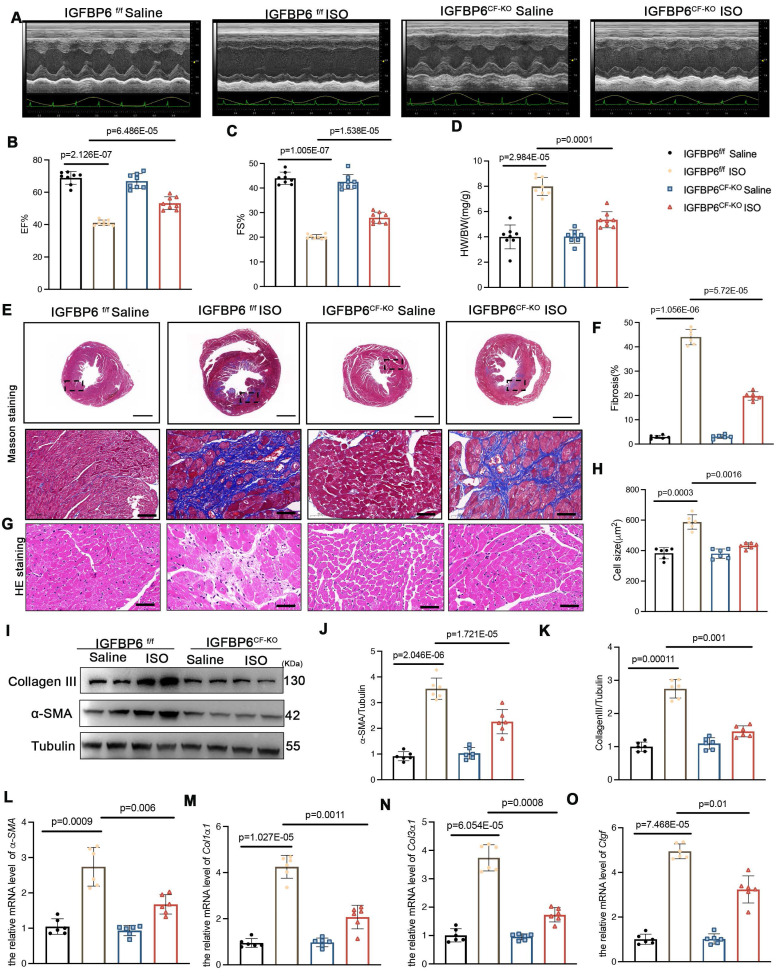
** Cardiac fibroblast-specific IGFBP6 deficiency attenuates ISO-induced cardiac fibrosis and cardiac dysfunction.** IGFBP6^f/f^ and IGFBP6^CF-KO^ mice were injected ISO (10 mg/kg/d) for 14 days. Cardiac function indices were measured by echocardiography. **A:** Representative M-mode echocardiographic images of IGFBP6^f/f^ and IGFBP6^CF-KO^ mice. **B-C:** Quantification of cardiac EF (ejection fraction) and FS (fractional shortening). n=8 mice per group.** D:** Quantification of HW/BW (heart weight/body weight) between IGFBP6^f/f^ and IGFBP6^CF-KO^ mice. n=8 mice per group. **E-F:** Representative images and quantification of heart sections stained with Masson trichrome staining at 2 weeks after ISO injection in IGFBP6^f/f^ and IGFBP6^CF-KO^ mice. n=6 mice per group. Scale bars: 500 µm and 50 µm. **G-H:** Representative images and quantification of cell size in left ventricular. n=6 mice per group. Scale bars: 50 µm. **I-K:** Western blot and analysis of α-SMA and Collagen III in the infarct zone at 2 weeks after ISO injection. n=6 mice per group. **L-O:** mRNA expression levels of α-SMA, Col1α1, Col3α1, Ctgf were detected by real-time quantification PCR. n=6 mice per group. Data are expressed as mean ± SD. Two-way ANOVA followed by Tukey's multiple comparisons test was used for analysis.

To validate these observations in an alternative model, we subjected mice to ISO-induced cardiac stress (10 mg/kg daily for 14 days). Echocardiography analysis confirmed that IGFBP6^CF-KO^ mice were protected from ISO-induced cardiac dysfunction, showing preserved EF and FS compared with IGFBP6^f/f^ controls (**Figure 4A-C, Supplementary Table S5**). IGFBP6 deficiency in the CFs significantly decreased the HW/BW, compared with IGFBP6^f/f^ mice with the ISO-challenge (**Figure 4D**). Furthermore, IGFBP6 deficiency mitigated ISO-triggered cardiomyocyte enlargement and interstitial fibrosis (**Figure 4E-H**). IGFBP6 knockout downregulated the *α*-SMA and collagen III expression levels in response to ISO challenge (**Figure 4I-K**). The mRNA expression of fibrosis-related genes was also decreased in IGFBP6^CF-KO^ mice (**Figure 4L-O**). These results demonstrates that IGFBP6 knockout in CFs effectively prevent cardiac remodeling in response to MI and ISO challenge.

### Myofibroblast-specific IGFBP6 knockout alleviates adverse remodeling and cardiac dysfunction after MI

**Figure 5:**
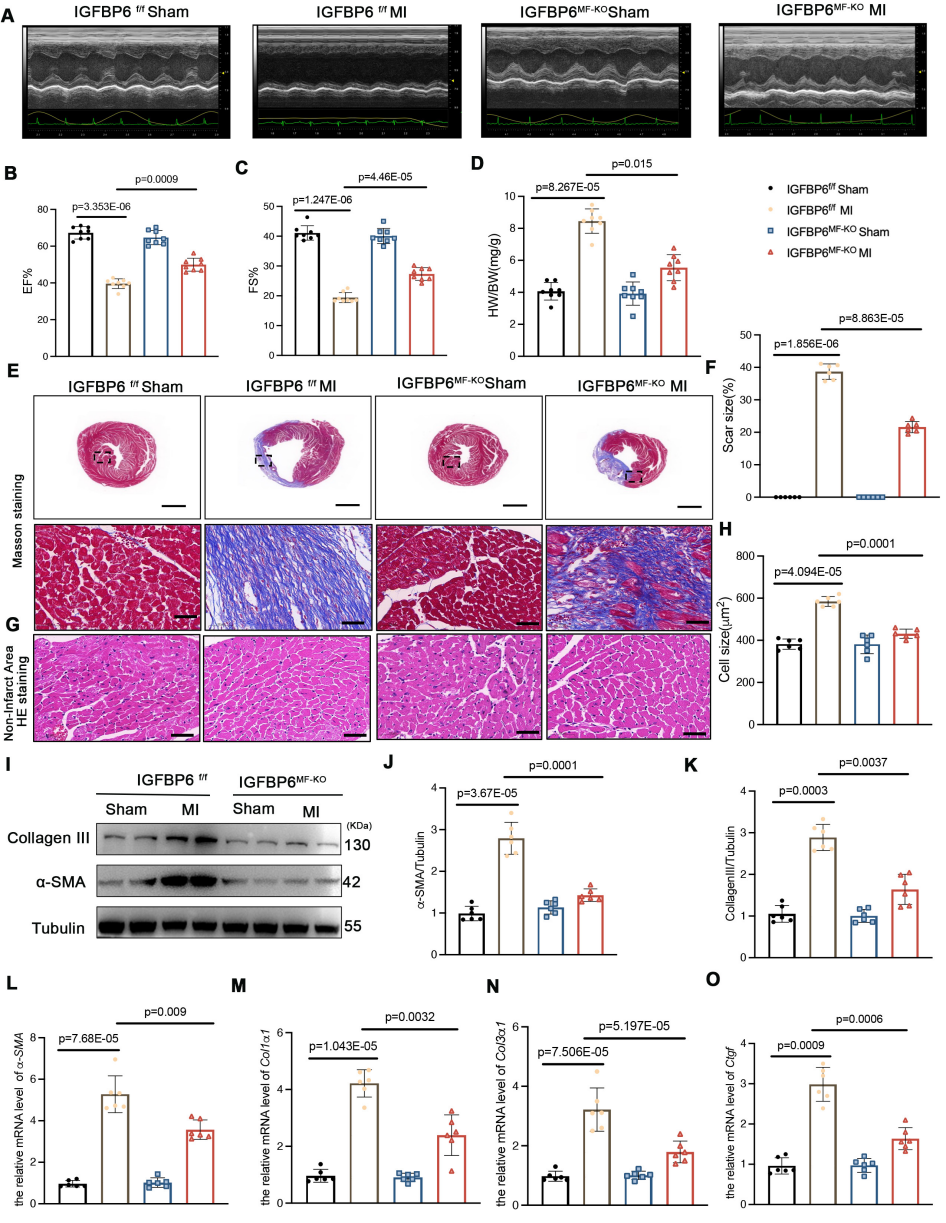
** Myofibroblast-specific IGFBP6 knockout attenuates adverse remodeling and cardiac dysfunction after MI. A-C:** IGFBP6^f/f^ and IGFBP6^MF-KO^ mice were injected tamoxifen for 5 continuous days after subjected to MI surgery for 7 days. At 14 days post-MI, cardiac function indices were measured by echocardiography. EF (ejection fraction) and FS (fractional shortening) were quantified. n=8 mice per group. **D:** Quantification of HW/BW (heart weight/body weight) between IGFBP6^f/f^ and IGFBP6^MF-KO^ mice. n=8 mice per group. **E-F:** Heart sections were stained with Masson trichrome at 2 weeks after MI. The scar size was measured and quantified as a percentage of total circumstance of left ventricle. n=6 mice per group. Scale bars: 500 µm and 50 µm. **G-H:** HE staining of cardiomyocyte cell size in left ventricular non-infarct zone. Scale bars: 500 µm and 50 µm. **I-K:** Western blot and analysis of α-SMA and Collagen III in the infarct zone at 2 weeks post-MI. n=6 mice per group. **L-O:** mRNA expression levels of α-SMA, Col1α1, Col3α1, Ctgf were detected by real-time quantification PCR. n=6 mice per group. Data are expressed as mean ± SD, two-way ANOVA followed by Tukey's multiple comparisons test was use for analysis.

**Figure 6:**
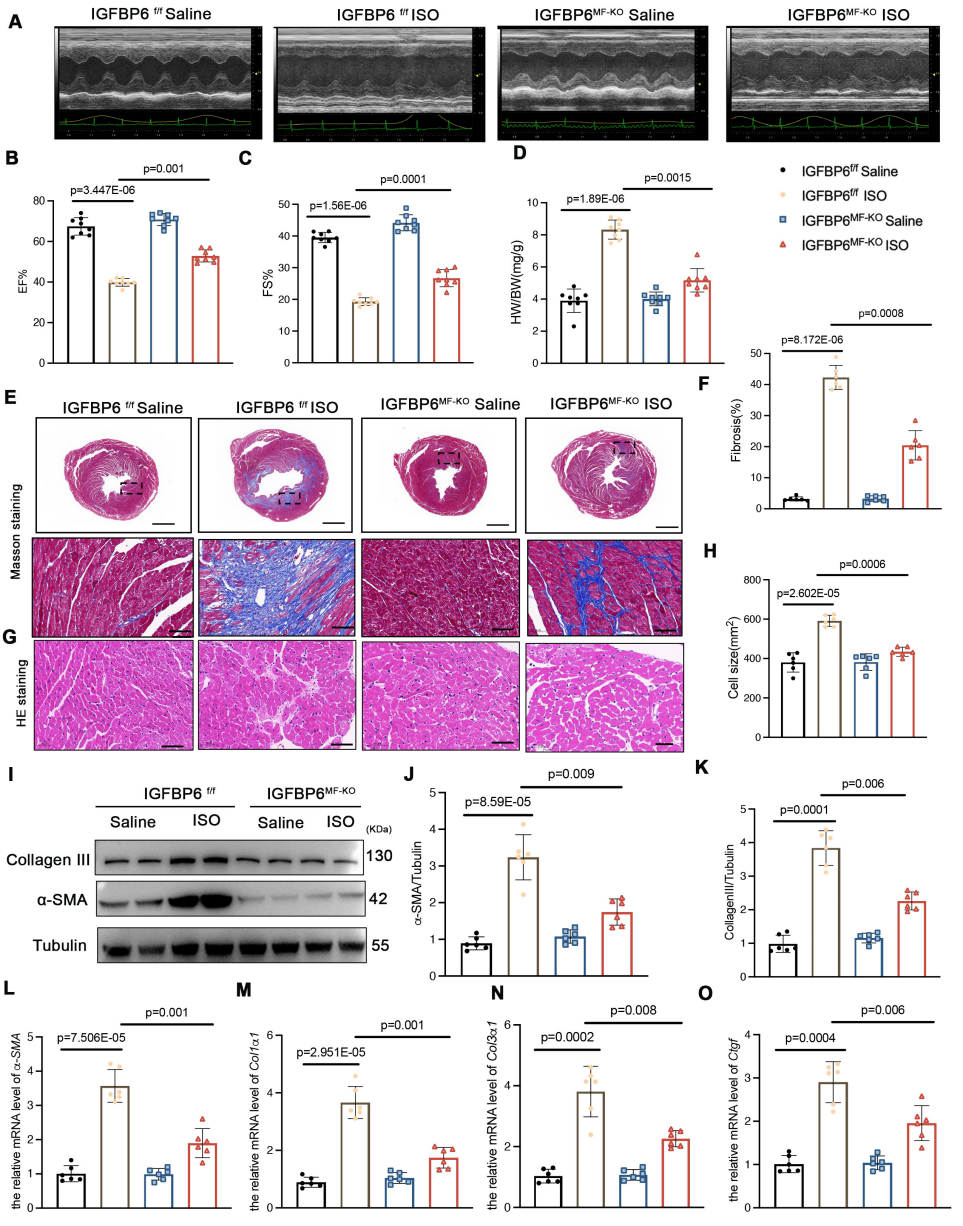
** Myofibroblast-specific IGFBP6 knockout alleviates ISO-induced cardiac fibrosis and cardiac dysfunction.** IGFBP6^f/f^ and IGFBP6^MF-KO^ mice were injected ISO (10 mg/kg/d) for 14 days. Cardiac function indices were measured by echocardiography. **A:** Representative M-mode echocardiographic images of IGFBP6^f/f^ and IGFBP6^MF-KO^ mice. **B-C:** Quantification of cardiac EF (ejection fraction) and FS (fractional shortening). n=8 mice per group.** D:** Quantification of HW/BW (heart weight/body weight) between IGFBP6^f/f^ and IGFBP6^MF-KO^ mice. n=8 mice per group. **E-F:** Representative images and quantification of heart sections stained with Masson trichrome staining in IGFBP6^f/f^ and IGFBP6^MF-KO^ mice. n=6 mice per group. Scale bars: 500 µm and 50 µm. **G-H:** Representative images and quantification of cell size in left ventricular. n=6 mice per group. Scale bars: 50 µm. **I-K:** Western blot and analysis of α-SMA and Collagen III in the fibrotic myocardium. n=6 mice per group. **L-O:** mRNA expression levels of α-SMA, Col1α1, Col3α1, Ctgf were detected by real-time quantification PCR. n=6 mice per group. Data are expressed as mean ± SD. Two-way ANOVA followed by Tukey's multiple comparisons test was used for analysis.

To delineate the temporal role of IGFBP6 during myofibroblast activation, we generated myofibroblast-specific IGFBP6 knockout mice (IGFBP6^MF-KO^) by crossing IGFBP6^f/f^ mice with Postn-MerCreMer mice (**Figure S4A**). The genotyping PCR results for IGFBP6^f/f^ mice and IGFBP6^MF-KO^ mice were shown **Figure S4B**. The IGFBP6^MF-KO^ mice were subjected to MI for 7 days to allow fibroblast-to-myofibroblast transition, followed by tamoxifen injection for 5 consecutive days starting on the 8^th^ day post-MI. We then isolated fibroblasts from MI hearts to evaluate the efficiency of the IGFBP6 knockout (**Figure S4C-F**). We then performed TTC staining at 7 days post-MI prior to initiating tamoxifen injection in order to exclude inherent differences related to the surgical procedures. We found no difference in myocardial infarct size between the IGFBP6^f/f^ and IGFBP6^MF-KO^ mice at 7 days post-MI prior to Cre activation (**Figure S4G-H**). The IGFBP6^MF-KO^ mice exhibited significantly preserved EF and FS after MI compared with the IGFBP6^f/f^ mice (**Figure 5A-C, Supplementary Table S6**). The IGFBP6^MF-KO^ mice showed lower HW/BW ratio post-MI than the IGFBP6^f/f^ mice (**Figure 5D**). Histological staining showed that IGFBP6 knockout in the myofibroblasts decreased the infarction size post-MI and suppressed adverse remodeling in the left ventricle (**Figure 5E-H**). Notably, IGFBP6 knockout in the myofibroblasts markedly ameliorated cardiac fibrosis as measured by the expression levels of pro-fibrogenic genes (**Figure 5I-O**). The effects of myofibroblast-specific IGFBP6 knockout on cardiac fibrosis and cardiac function were verified in an ISO-induced cardiac fibrosis model (**Figure 6, Supplementary Table S7**). We found that IGFBP6 knockout mitigated cardiac fibrosis and boosted heart function with ISO challenge. Collectively, these data suggest that IGFBP6 knockout in the myofibroblasts may be an effective strategy to reverse cardiac remodeling after cardiac injury.

### IGFBP6 interacts with EGR1 to activate MFAP4 transcription in CFs

**Figure 7:**
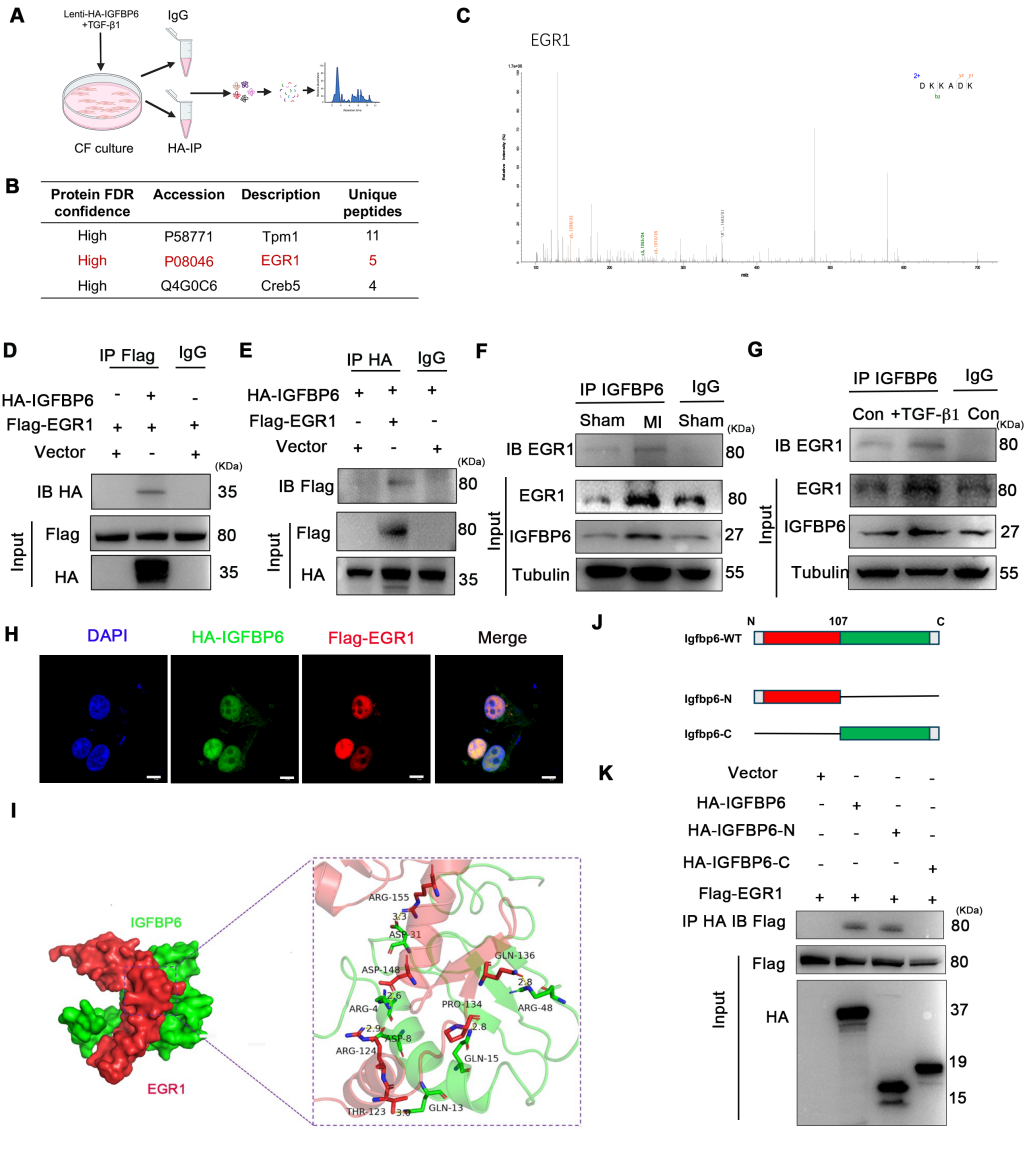
** IGFBP6 interacts with EGR1 via the N-terminal domain. A:** Schematic illustration of quantitative proteomic screen to identify proteins binding to IGFBP6. **B:** Lysates from CFs were immunoprecipitated with anti-IGFBP6 antibody followed by mass spectrometry analysis to identify the proteins that interact with IGFBP6. The graph showed the potential related proteins. **C:**MS/MS spectrum of the peptide showing DKKADK from EGR1. Single-letter abbreviations: D, Asp; K, Lys; K, Lys; A, Ala; D, Asp; K, Lys. **D-E:** HEK293T cells were transfected for 24 hours with plasmids encoding either Flag-EGR1 or HA-IGFBP6 alone or in combination. Cell lysates were immunoprecipitated with Flag and HA antibodies and immunoblot assays were performed using HA or Flag antibodies. **F-G:** Lysates of MI heart tissues and cell lysates of CFs were immunoprecipitated with IgG or IGFBP6 antibodies, and immunoblot assays were performed using EGR1 antibody. **H:** Triple immunofluorescence (IF) staining for HA-IGFBP6 (green) and Flag-EGR1 (red) and nuclei (DAPI, blue) was performed in HEK293T cells. Scale bars: 5 µm **I:** Surface diagram of the docking model and their interfacing residues between IGFBP6 with EGR1 proteins (IGFBP6 green; EGR1 red; hydrogen bond interaction, dotted line). **J:** Schematic representations of IGFBP6 residues and domains of IGFBP6 involved in binding to EGR1. **K:** HA-tagged-full-length IGFBP6 or deletion of domains of IGFBP6 and full-length of EGR1 were constructed and then transfected into HEK293T cells. Immunoprecipitated with HA antibody, and immunoblot assays were performed using Flag antibody.

To further explore the molecular mechanism by which IGFBP6 regulated cardiac remodeling, we performed co-immunoprecipitation (CO-IP) combined with mass spectrometry (MS) analysis in CFs transfected with HA-tagged IGFBP6 (Lenti-HA-IGFBP6) or EGFP control (Lenti-HA-EGFP). MS analysis of IGFBP6 immunocomplexes revealed peptides corresponding to EGR1, suggesting a potential interaction between these two proteins (**Figure 7A-C**). To validate this interaction, we co-transfected HEK293T cells with HA-IGFBP6 and Flag-EGR1 expression vectors. Immunoprecipitation assays demonstrated a direct physical association between IGFBP6 and EGR1, as evidenced by reciprocal pulldown of both proteins (**Figure 7D-E**). Notably, this interaction was significantly enhanced under both the pathological conditions, MI *in vivo* and TGF-β1 treatment *in vitro* (**Figure 7F-G**). Immunofluorescence staining assay revealed that IGFBP6 and EGR1 were mainly colocalized in the nucleus of HEK293T cells (**Figure 7H**). Structure modeling using Z-DOCK predicted specific molecular contacted stabilizing the IGFBP6-EGR1 complex. Key hydrogen bonds were identified between IGFBP6 residues (GLN-13, ASP-8, ARG-4, GLN-15, ARG-48, ASP-31) and EGR1 residues (THR-123, ARG-124, ASP-148, PRO-134, GLN-136, ARG-155), suggesting that these residues formed the interaction interface (**Figure 7I**). To determine which domain of IGFBP6 might interact with EGR1, we constructed HA-tagged full length IGFBP6 (1-240aa, HA-IGFBP6-WT), HA-IGFBP6 with an N-terminal domain (1-107aa, HA-IGFBP6-N), and HA-IGFBP6 with a C-terminal domain (108-240aa, HA-IGFBP6-C) with a pCDNA-3
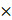
HA-EGFP vector. The domain boundaries for IGFBP6 truncations were defined based on UniProt-predicted domains (28-107aa and 160-234aa) and protein identifying EGR1-binding residues within 1-107aa. We also constructed Flag-EGR1 with a pEn-CMV-3
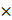
Flag vector and then transfected them into HEK293T cells. As shown in **Figure 7J-K**, the interaction with EGR1 was exclusively eliminated in cells overexpressing HA-IGFBP6-C.

**Figure 8:**
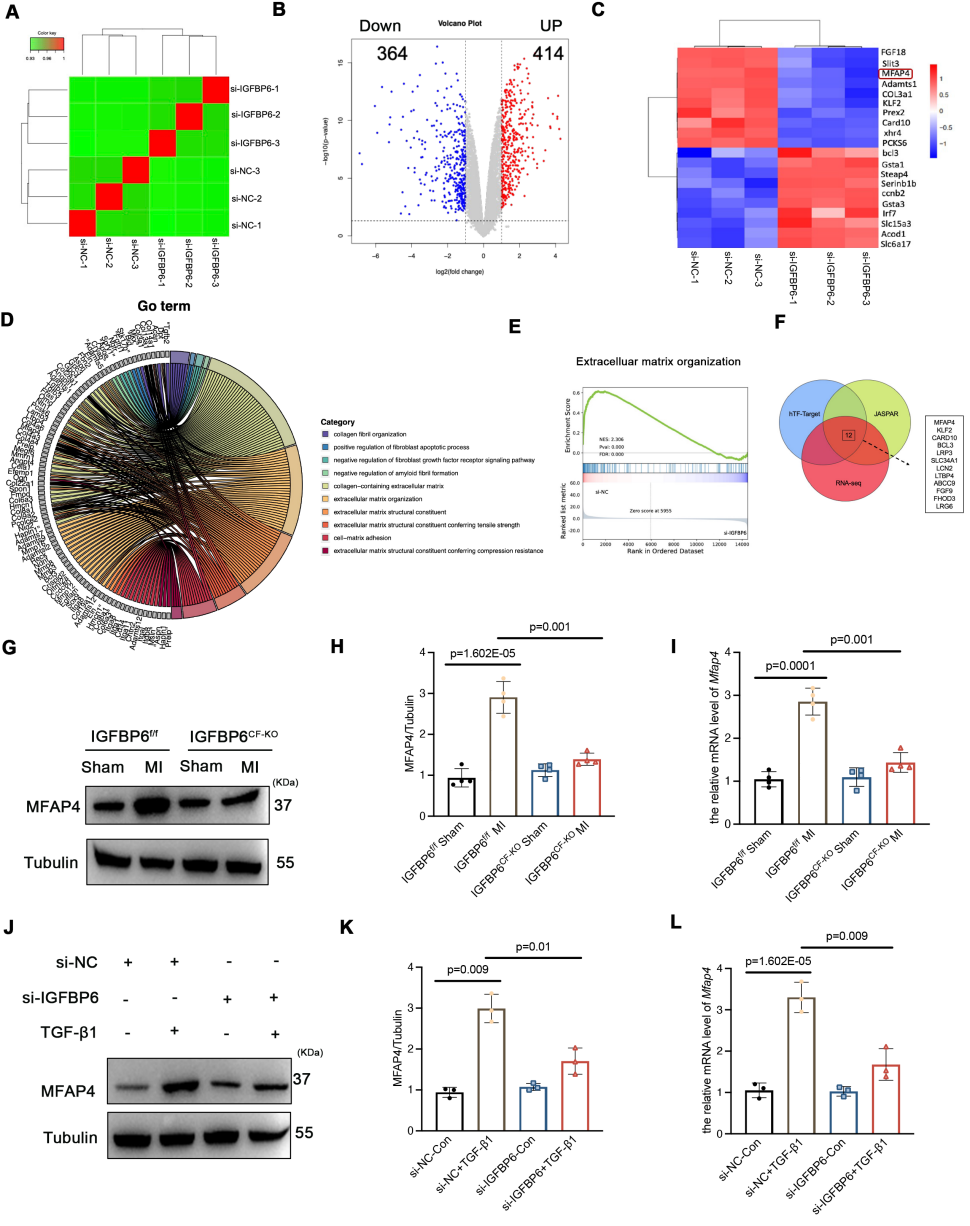
** IGFBP6 regulates extracellular matrix organization via upregulates the MFAP4 expression.** Mouse CFs were transfected with si-IGFBP6 or si-NC followed by exposure to TGF-β1 (10 ng/mL) for 48 h. RNA-Seq analysis was performed to reveal differentially expressed genes (DEGs). **A:** Hierarchical clustering diagram of gene expression levels of samples. The color scale from green to red represents low and high inters ample correlations obtained by gene expression levels. **B:** Volcano plot.** C:** Heatmap of differentially expressed genes. **D:** Go Analyze the sine curve** E:** Gene Set Enrichment Analysis (GSEA).** F:** Venn diagram showing the potential transcription factors for MFAP4 which was target gene of EGR1.** G-I:** The protein and mRNA level of MFAP4 in IGFBP6^f/f^ and IGFBP6^CF-KO^ mice MI myocardium. n=6 mice per group. **J-L:** The protein and mRNA level of MFAP4 in si-IGFBP6 or si-NC followed by exposure to vehicle or TGF-β1. n=3 independent biological. Data are expressed as mean ± SD. Two-way ANOVA followed by Tukey's multiple comparisons test was used for analysis.

We also treated the CFs with TTGF-β1 after silencing IGFBP6 and then performed RNA-Seq analysis to evaluate the differential expression of genes (DEGs). RNA-seq results showed that there were 414 upregulated and 364 downregulated DEGs between the si-NC + TGF-β1 and si-IGFBP6 + TGF-β1 groups (|log2(FC)| > 1.5, p value < 0.05) (**Figure 8A-B**). Among these DEGs, we identified the top 10 genes that were upregulated and downregulated by IGFBP6 knockdown (**Figure 8C**). GO and GSEA analyses revealed that IGFBP6 influenced the transcription of genes involved in ECM production, ECM organization, and cell-matrix adhesion (**Figure 8D-E**). We then employed an integrated computational approach by comparing the JASPAR database, hTFtarget database, and data from our RNA-sequencing analysis and found that MFAP4 was the top-ranked gene among the 10 most significant downregulated targets (**Figure 8F**). It has been reported that MFAP4 is upregulated in fibrotic hearts and that the loss of MFAP4 attenuates Ang-II-induced left atrial fibrosis 24. As shown, IGFBP6 knockout in CFs downregulated the mRNA and protein expression of MFAP4 post-MI (**Figure 8G-I**). In addition, the protein and mRNA expression of MFAP4 were upregulated by TGF-β1 treatment in an IGFBP6-dependent manner (**Figure 8J-L**).

**Figure 9:**
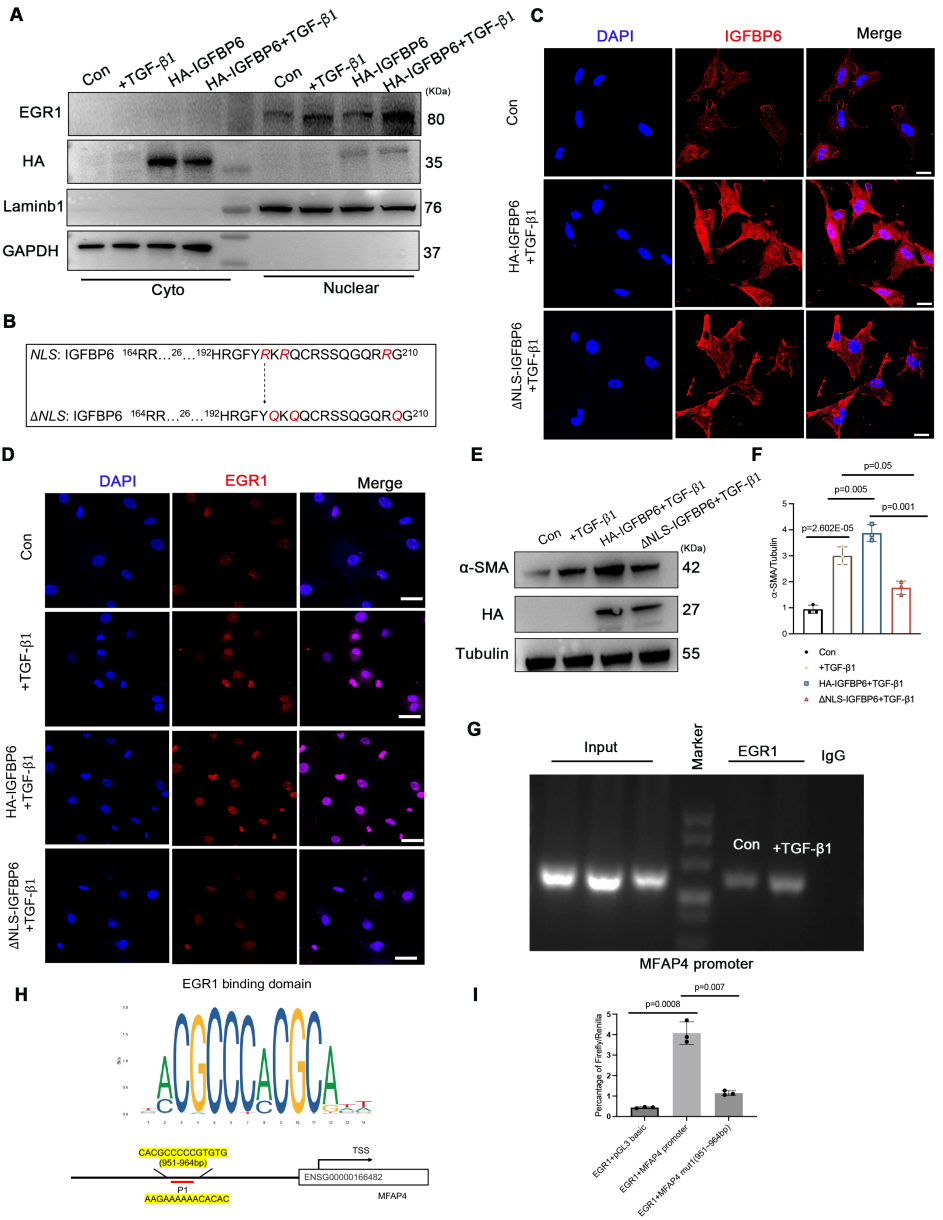
** IGFBP6 translocate into nucleus via its NLS-sequence and promotes EGR1-mediated MFAP4 transcription. A**: WT CFs were transfected with HA-IGFBP6 lentivirus and performed TGF-β1 challenge (10 ng/mL, 48 h). Cytoplasmic and nuclear proteins were isolated. Western blot analysis was performed to detect the expression of EGR1. n=3 independent biological cultures. **B:** Schematic illustration of △NLS-IGFBP6 construction. CFs were transfected with HA-IGFBP6 or HA-△NLS-IGFBP6 lentivirus and challenged with TGF-β1. **C:** Immunofluorescence staining for IGFBP6 (red) and nuclei (DAPI, blue) was performed in CFs. Scale bars: 20 µm. n=3 independent biological cultures. **D:** Immunofluorescence staining for EGR1 (red) and nuclei (DAPI, blue) was performed in CFs. n=3 independent biological cultures. **E-F:** Cell lysates were examined by immunoblot analysis to detect the expression of α-SMA. n=3 independent biological cultures. **G:** The independent CH-IP assay was performed on Lenti-Scramble or Lenti-Flag-EGR1-infected CFs to verify EGR1 binding to the promoter regions of MFAP4. n=3 independent biological cultures. **H:** Schematic diagram of the construction of wild type and mutant luciferase reporter plasmids of MFAP4 promoter. **I**: Luciferase activation driven by the wild type or mutant promoter of MFAP4 after normalization to Renilla luciferase in HEK293T cells. n=3 independent biological cultures. Data are shown as mean ± SD. Significance was assessed by one-way ANOVA or two-way ANOVA and Tukey post hoc test.

We then overexpressed HA-IGFBP6 in the CFs with the stimulation of TGF-β1 and detected the expression of EGR1 in the nucleus. As shown, overexpression of IGFBP6 further increased the accumulation of EGR1 in the nucleus (**Figure 9A**). It has been shown that IGFBP6 contains both a nuclear translocation signal (NLS) and a transactivation domain that enable IGFBP6 to migrate into the nucleus in an IGF-II independent manner 25. TGF-β1 stimulation indeed promoted the nuclear translocation of IGFBP6 in CFs. We then constructed HA-tagged WT-IGFBP6 and HA-tagged △NLS-IGFBP6 (**Figure 9B-C**) lenti-virus and then infected the CFs with the TGF-β1 stimulation. As shown, WT-IGFBP6, but not △NLS-IGFBP6, augmented TGF-β1-induced EGR1 expression in the CFs (**Figure 9D**). In addition, △NLS-IGFBP6 could not regulate the expression of cardiac fibrosis genes with TGF-β1 stimulation (**Figure 9E-F**). These results suggest that the translocation of IGFBP6 into the nucleus is dependent on its NLS sequences and the translocated IGFBP6 then regulates myofibroblasts transition.

We next explored whether IGFBP6 regulated the expression of MFAP4 through EGR1. EGR1 is well-recognized as a transcription factor that modulates gene transcription. To further investigate the interaction between EGR1 and MFAP4 promoter, we conducted chromatin immunoprecipitation assay in CFs infected with Lenti-EGFP or Lenti-Flag-EGR1 (**Figure 9G**). In addition, we identified the specific binding sites between EGR1 and MFAP4 using the JASPR database to predict candidate binding sites (**Figure 9H**). The sequences of MFAP4 were cloned into PGL3-based luciferase reporter plasmids (named P1), which were then transfected into EGR1-overexpressed HEK293T cells. Luciferase assays were subsequently performed by cloning mutated -951 to -964bp fragments of the MFAP4 promoter (Mut-MFAP4). As shown in **Figure 9I**, Mut-MFAP4 abrogated the putative EGR1-binding sites, and WT fragments sequences were used as control. Taken together, our data indicate that IGFBP6 interacts with EGR1 to activate MFAP4 transcription in CFs.

### MFAP4 is essential for the regulatory role of IGFBP6 in cardiac ischemic injury post-MI

**Figure 10:**
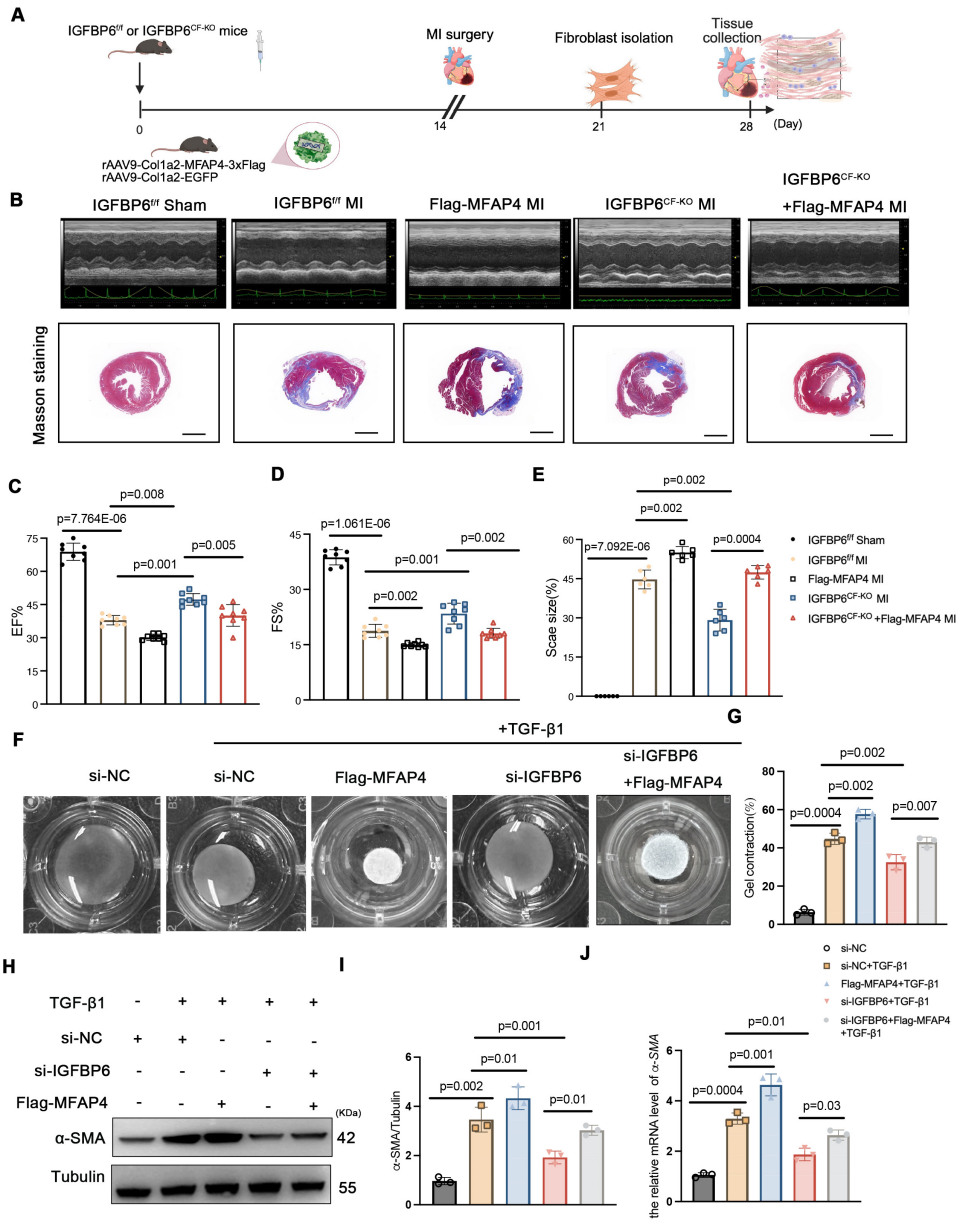
** The regulatory role of IGFBP6 in cardiac ischemic injury post-MI depends on MFAP4. A:** IGFBP6^f/f^ and IGFBP6^CF-KO^ mice were injected with rAAV9-Col1a2-EGFP in the presence or absence of rAAV9-Col1a2-MFAP4-3
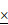
Flag before they subjected to MI. Protocol and schematics of rAAV9-virus injection and MI surgery. **B:** M-mode echocardiography and Masson staining for mice at day 14 post-MI. **C-D:** Quantification of cardiac EF (ejection fraction) and FS (fractional shortening). n=8 mice per group.** E:** Masson trichrome staining and the quantification of mice at 2 weeks after MI. n=6 mice per group. Scale bars: 500 µm. **F-G:** WT cardiac fibroblasts were infected with si-IGFBP6 with or without lentivirus-Flag-MFAP4 and lentivirus-EGFP. After infected with 48 h, CFs were treated with TGF-β1 (10 ng/mL, 48 h). Collagen contractility with representative collagen gels showing contraction 48 h after the gel release, with percent collagen gel contraction quantified over 48 h period. n=3 independent biological cultures. **H-J:** Cell lysates were examined by immunoblot and qRT-PCR analysis to detect the expression of α-SMA. n=3 independent biological cultures. Data are expressed as mean ± SD. Two-way ANOVA followed by Tukey's multiple comparisons test was used for statistical analysis.

To explore whether IGFBP6 regulated adverse remodeling and myofibroblasts transition via MFAP4, we administered rAAV9-Col1a2-MFAP4-3
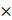
Flag to IGFBP6^f/f^ and IGFBP6^CF-KO^ mice through tail vein injection. A brief flow chart of this experiment is presented in **Figure 10A**. We observed a notable elevation in MFAP4 protein levels in the left ventricular myocardium two weeks after injection (**Figure S5B**). Immunofluorescence staining of heart tissues with anti-flag antibody revealed stronger positive MFAP4 staining in fibroblasts within heart tissues overexpressing MFAP4 (**Figure S5C**). Transfection with rAAV9-Col1a2-MFAP4-3
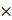
Flag did not affect the cardiac function of IGFBP6^CF-KO^ mice without surgery, as indicated by the EF and FS values. Echocardiographic analysis indicated that fibroblasts-specific knockout of IGFBP6 increased EF and FS in mice subjected to MI surgery, but the protective effects of IGFBP6 knockout in CFs were abolished when MFAP4 was overexpressed (**Figure 10B-E, Supplementary material online Table S8**). We then transfected the CFs with si-IGFBP6, followed by infection with or without Lentiviral-Flag-MFAP4 upon TGF-β1 stimulation. As shown, the protective effects of silencing IGFBP6 were markedly abolished in cells infected with Lentiviral-Flag-MFAP4 (**Figure 10F-J**). Overexpression of MFAP4 significantly increased the protein and mRNA expression levels of α-SMA in the IGFBP6 knockdown CFs (**Figure 10H-J**). These results collectively demonstrate that IGFBP6 contributes to cardiac remodeling and cardiac dysfunction, at least in part, through the activating of MFAP4.

## Discussion

Adverse cardiac remodeling, characterized by excessive collagen deposition, fibrosis, and subsequent cardiac dysfunction, is a critical precursor to heart failure. Dysregulation of cardiac fibroblasts (CFs) plays a central role in this process, yet the molecular mechanisms governing their transition to hyperactive myofibroblasts are not fully understood. Addressing this knowledge gap is essential for developing targeted therapies to halt disease progression 26. This study identifies insulin-like growth factor binding protein 6 (IGFBP6) as a pivotal pro-fibrotic factor in both murine and human fibrotic myocardium. Notably, IGFBP6 exerts its effects through an IGF-II-independent pathway—a previously unrecognized mechanism. Utilizing multimodal mechanistic analyses, we demonstrate that IGFBP6 translocate into the nucleus via its nuclear localization sequences (NLS) and directly interacts with early growth response protein 1 (EGR1). This interaction enhances EGR1 binding to the promoter of microfibril-associated protein 4 (MFAP4), a key mediator of ECM remodeling. Furthermore, overexpression of MFAP4 significantly reverses the protective effects of knockout IGFBP6 in the fibroblasts-to-myofibroblast transition (**Graphical Abstract**). This rescue experiment establishes MFAP4 as a downstream effector in the IGFBP6/EGR1 signaling axis. Our findings delineate a novel IGFBP6-EGR1-MFAP4 signaling pathway that drives pathological myofibroblast-transition. The discovery of the nuclear localization and transcription-regulatory functions of IGFBP6 expands its known roles beyond extracellular IGF signaling. These results position IGFBP6 as a promising therapeutic target to disrupt maladaptive cardiac remodeling and prevent heart failure progression.

IGFBPs are characterized by a conserved modular structure, featuring cysteine-rich N-terminal domain and C-terminal domain that are stabilized by intradomain disulfide bonds. These structural motifs confer rigidity to the terminal regions and are critical for high-affinity IGF binding 27. Beyond their canonical role in IGF sequestration, accumulating evidence highlights the intrinsic IGF-independent functions of IGFBPs, including modulation of cell growth, apoptosis, and transcriptional regulation 28. Notably, IGFBPs have emerged as key regulators in cardiovascular pathophysiology 29-31. For instance, IGFBP5 knockdown attenuated cardiac fibrosis in murine models 32. IGFBP7 accelerated the progression of heart failure and cardiac fibrosis by suppressing FOXO3a via the IGF-1R/IRS/AKT axis 33. In the present study, we identified significant upregulation of IGFBP6 in three distinct cardiac fibrosis models (myocardial infarction, isoproterenol-induced injury, and transverse aortic constriction). RNA-seq data from ischemic cardiomyopathy patients (GSE145154) further revealed enriched IGFBP6 expression specifically in CFs, but not in endothelial cells and other cell types. In the present study, we found that IGFBP6 was significantly increased in the infarcted myocardium and CFs at 14 days post-MI. In addition, we found that serum IGFBP6 levels were increased in both human and murine MI models. Following ischemic injury, stressed or infarcted cardiomyocytes and activated CFs may release IGFBP6 into the circulation as part of the systemic response to tissue damage. These findings collectively implicate IGFBP6 as a potential mediator of fibrotic remodeling, yet its mechanistic contributions to heart failure pathogenesis warrant further investigation.

Previous studies have established IGFBP6 as a critical regulator of cellular processes, including cell cycle progression and cell proliferation and differentiation 15. While existing evidence indicates reduced IGFBP6 expression during the acute phase of MI 34, our investigation reveals a temporal dichotomy in its post-MI regulation. Specifically, quantitative protein analysis demonstrated a progressive elevation of IGFBP6 from day 3 to day 14 post-MI when compared with the sham group, contrasting with a transient mRNA downregulation at 1-day post-MI that lacked the corresponding protein-level significance. This discordance is further reflected in serum profiling, showing acute-phase depletion (1-day) followed by chronic-phase elevation (14-day). We hypothesize that non-coding RNA-mediated post-transcriptional regulation may impair mRNA stability and translational efficiency during early MI. Notably, our functional studies implicate IGFBP6 in orchestrating dual-phase immune responses: early-phase inflammatory activation through TLR4 pathway potentiation, and chronic-phase fibro-inflammatory recruitment during cardiac repair. This context-dependent functionality aligns with emerging evidence supporting the role of IGFBP6 in tissue-specific inflammatory modulation. *Su et al.* reported that endothelial cell (EC)-specific overexpression of IGFBP6 ameliorates atherosclerosis and endothelial cell inflammation by inhibiting the nuclear factor kappa B (NF-κB) pathway 35. *Lucia et al.* has reported that IGFBP6 can promote the activation of downstream TLR4 signal and immune inflammatory responses, thereby aggravating myelofibrosis 17. IGFBP6 is significantly positively associated with steatosis and has been identified as a contributor to hepatic inflammation and fibrosis 36. These paradoxical findings suggest a cellular origin-dependent paradigm where IGFBP6 inflammatory impact is determined by the secreting cell types, tissue microenvironment, and disease stage. The observed cardiac-specific temporal regulation may reflect compartmentalized functions-early depletion permitting initial inflammatory responses, followed by sustained elevation mediating fibrotic repair.

The transcription factor EGR1 serves as a master regulator of fibroblasts activation in adverse remodeling 37. *Zhang et al.* demonstrates that EGR1 activates the integrin-FAK-ERK-Akt1 axis in CFs 38. Our immunofluorescence and subcellular fractionation data revealed that IGFBP6 translocated to the nucleus via NLS signaling upon TGF-β1 stimulation in the MI infarction areas. This nuclear localization strongly suggested a transcriptional regulatory role for IGFBP6 in fibrotic pathways. Its nuclear activity aligned with the observed nuclear localization of IGFBP6 as well as it focused on the transcriptional regulation of MFAP4. While Tropomyosin 1 (Tpm1) was identified in our IP/MS analysis, it primarily localized to the cytoplasm and regulated cytoskeletal dynamics and muscle contraction. Its role in cytoskeletal stabilization was less directly connected to the nuclear/transcriptional mechanisms central to our study. Building on its foundation, our study uncovers a spatiotemporal coordination mechanism involving IGFBP6 and EGR1.

Leveraging subcellular fractionation assays, we observed that IGFBP6, which was previously been reported to exhibit dual cytoplasmic/nuclear localization, undergoes NLS-dependent nuclear translocation post-MI 25. Nuclear uptake and accumulation of IGFBP-6 might change the cell fate toward apoptosis or also induce differentiation in muscle cells 39. Co-immunoprecipitation and domain mapping revealed direct physical interaction between the cysteine-rich N-domain of IGFBP6 and EGR1, forming a transcriptional complex that bound to the MFAP4 promoter. Emerging evidence highlights the pivotal role of MFAP4 in fibrotic pathogenesis. Our study identifies MFAP4 as a novel downstream effector of the IGFBP6-EGR1 signaling axis, driving fibroblast-to-myofibroblast transition and cardiac fibrosis. This finding aligns with recent reports demonstrating that MFAP4 deletion attenuates Ang II-induced atrial fibrosis and atrial fibrillation in murine models, suggesting its dual role in fibrotic remodeling 24. In addition, MFAP4 is primarily expressed by myofibroblasts originating from cardiac fibroblasts 40. Our data provide the direct evidence that fibroblast-specific MFAP4 overexpression counteracts the anti-fibrotic effects of IGFBP6 knockout, restoring collagen deposition and impairing cardiac function in mice. This regulatory interplay may involve the recently characterized octameric structure of MFAP4, which facilitates multivalent interactions with elastogenic proteins such as fibrillin-1 and tropoelastin. Such structural features might amplify the capacity of MFAP4 to stabilize pro-fibrotic signaling complexes at the cell-matrix interface. Importantly, the calcium-dependent oligomerization of MFAP4 observed in cryo-EM studies could dynamically regulate its bioavailability during fibrotic progression, a hypothesis that warrants further investigation. MFAPs play important roles in cholesterol synthesis in CFs by promoting SREBP2 maturation 41-43. In addition, proteomic analysis of cells with knockdown of the IGFBP6 gene reveals a significant group of proteins involved not only in the biosynthesis and transport of fatty acids but also in other vital cellular processes, showing reduced expression relative to the control cell lines 44. IGFBP6 may also aggravate myocardial fibrosis by regulating SENP2 maturation of myocardial fibroblasts, thereby regulating cholesterol flow.

This study also has limitations that warrant further consideration. First, while we elucidated the critical role of IGFBP6 in cardiac fibroblasts and myofibroblasts during adverse remodeling in chronic myocardial infarction, the origin and functional implications of elevated IGFBP6 levels in the peripheral blood remain uncharacterized. Second, although our findings suggest multiple potential mechanisms through which IGFBP6 may influence post-MI recovery, the relative contribution of these pathways to cardiac functional restoration requires systematic comparative analysis. Third, the exclusive use of male mice represents a significant constraint, as sexual dimorphism in cardiovascular pathophysiology is well-documented-particularly given that human epidemiological data exclude potential sex-dependent regulation of IGFBP6 functions. Therefore, our findings cannot exclude potential sex-dependent regulation of IGFBP6 functions.

In summary, our study provides new insights into the regulatory mechanism of IGFBP6 in regulating adverse remodeling after MI and other stimulations. Moreover, these observations further verify MFAP4 as a downstream target gene of EGR1. These findings not only deepen our understanding of cardiac fibrosis but also highlight IGFBP6 as a potential therapeutic node for modulating maladaptive remodeling processes. Further investigations should prioritize sex-stratified analyses and translational validation of circulating IGFBP6 as a clinical biomarker.

## Supplementary Material

Supplementary methods, figures and tables.

## Abbreviations

IGFBP6): Insulin-like growth factor-binding protein 6
IGFs): insulin-like growth factors
GEO): Gene Expression Omnibus
MI): myocardial infarction
FMT): fibroblast-to-myofibroblast transition
EGR1): early growth regulator 1
MFAP4): microfibril-associated protein 4
ECM): extracellular matrix
CFs): Cardiac fibroblasts
NAFLD): non-alcoholic fatty liver disease
TLR4): Toll-like receptor 4
SHH): Sonic Hedgehog
NLS): nuclear localization signal
PCI): percutaneous coronary intervention
DMEM): Dulbecco's Modified Eagle Medium
FBS): Fetal Bovine Serum
siRNA): Small interfering RNA
ChIP): Chromatin Immunoprecipitation
NF-κB): nuclear factor kappa B
