# Supplementary material for: Cardiac fibroblast-derived IGFBP6 orchestrates cardiac remodeling by coupling the EGR1-MFAP4 axis
Source: Int J Biol Sci. 2025 Oct 1;21(14):6430–51. doi: 10.7150/ijbs.114417 (PMC12594604; doi:10.7150/ijbs.114417)
Supplement: Supplementary file 1 — Supplementary methods, figures and tables. [file ijbsv21p6430s1.pdf]

Supplementary material

Cardiac fibroblast-derived IGFBP6 orchestrates cardiac remodeling by coupling the EGR1-MFAP4 axis

Materials and Methods

Antibodies and Reagents

All primary antibodies against the indicated proteins used for immunoblotting, immunoprecipitation and immunostaining analysis in the present study are listed as follows: IGFBP6 (DF8959, Affinity, USA), α-SMA (A2547, Sigma-Aldrich, USA), Tubulin (Abcam, ab6046), β-actin (Abcam, ab6276), HA (#3724, CST, USA), Flag (F9291, Sigma-Aldrich, USA), His (#2365, CST, USA), P-Smad2/3 (#8828, CST, USA), T-Smad2/3 (#8685, CST, USA), EGR1 (Abcam, ab300449), Laminb1 (Abcam, ab16048), GAPDH (Abcam, ab8245), MFAP4 (BD, PA5-24865), Vimentin (Abcam, ab8978), Tubulin (Beyotime, AF2835). Alexa Fluor® 555, 647 and 488 conjugated secondary antibodies were purchased from Abcam. TGF-β1 (cat# 100-21, PeproTech, Rocky Hill, NJ, USA).

Generation of knockout of fibroblasts and myofibroblast mice

Conditional transgenic mice targeting IGFBP6 gene (IGFBP6$^{f/f}$) were generated by GemPharmatech (Nanjing, China) in the C57BL/6J background. Briefly, loxp sites were inserted on both sides of Exon1 to induce ATG deletion in Exon1, causing IGFBP6 to lose its protein-coding function. The Col1a2-Cre/ERT mice, containing a Cre-ERT recombinase gene, were purchased from Cyagen Biosciences Inc (#C001026). IGFBP6$^{f/f}$ mice were bred with Col1a2-Cre/ERT mice to achieve fibroblast-specific IGFBP6 knockout in the mice (IGFBP6$^{CF\text{-}KO}$). IGFBP6$^{f/f}$ mice were bred with Postn-Cre/ERT mice to achieve myofibroblast-specific IGFBP6 knockout in the mice (IGFBP6$^{MF\text{-}KO}$). Mice were intraperitoneally treated with 30 mg/kg of tamoxifen dissolved in corn oil for 5 days at 8 weeks of age. IGFBP6$^{f/f}$ mice tamoxifen littermates were represented as controls. Mice of different strains were genotyped with PCR.

Primers were as follows: 5’-CTGCTGTCCATTCCTTATTCCATAG-3’(sense) and 5’-CTGGAAATCAGGCTGCAAATCTC-3’(antisense), which yielded 233 bp products for IGFBP6$^{f/f}$. Col1a2-Cre was genotyped by the primers as follows: 5’- TCCAATTTACTGACCGTACACCAA-

3'(sense) and 5'- CCTGATCCTGGCAATTTCGGCTA -3'(antisense), which yielded 500 bp products. Postn-Cre was genotyped by the primers as follows: 5'-GGTGGGACATTTGAGTTGCT-3'(sense) and 5'- CCTTGCAATAAGTAAAACAGCTC -3'(antisense), which yielded 268 bp products.

Echocardiography Analysis

Cardiac function was measured at baseline before MI or sham surgery and 2 weeks post-MI. Cardiac function was assessed by transthoracic echocardiography (Visual Sonics Vevo 2100 Imaging System, Toronto, Canada) equipped with a 30-MHZ linear transducer. Mice were anesthetized with isoflurane (1.5% with O_2 1 L/min) and subsequently placed on a heated pad to sustain body temperatures within the range of 36.9°C to 37.3°C. The heart rates were maintained at around 500 bpm. M-mode echocardiography was performed by a Left ventricles (LV) were fully viewed by the results of 3 continuous heartbeats. LV end-systolic diameters (LVESD), LV end-diastolic diameters (LVEDD), LV end-systolic volume (LVESV), and LV end-diastolic volume (LVEDV) were measured. LV ejection fractions (LVEF) and LV fractional shortenings (LVFS) were calculated. The subsequent analysis was conducted by an investigator who was blind to the allocation of treatment and genotype.

2,3,5-triphenyltetrazolium chloride (TTC) staining and the infarction size measurement

IGFBP6$^{f/f}$ mice and IGFBP6$^{CF\text{-}KO}$ mice were subjected to MI surgery. IGFBP6$^{f/f}$ mice and IGFBP6$^{MF\text{-}KO}$ mice were also subjected to MI surgery. After 3 days or 7 days post-MI, the hearts were rapidly removed from the mouse and washed by ice-old PBS. The heart was then frozen for 15 min on the solidified carbon dioxide and cut into 1 mm thick slices along the atrioventricular sulcus from the apex to the bottom. The heart was cut into four to five pieces. The pieces were quickly placed into 5 mL 0.5% TTC buffer (pH = 7.4) for 15 min at 37°C. TTC staining showed that the infarcted area was white, and the non-infarcted area was red. The white/red area ratio at the papillary muscle surface was calculated by ImageJ software and recognized as the infarction size.

Masson trichrome staining

After 14 days post-MI, mice myocardium tissues were fixed with 4% paraformaldehyde for 24 h, dehydrated by increasing concentrations of ethanol, and embedded in paraffin. Tissue sections (5 μm) were stained with Masson trichrome staining kit (Sigma Aldrich, Shanghai, China) as the manufacturer's instruction described**.** The fibrotic area in each field was quantified using NIH ImageJ software. For quantification, total fibrosis was averaged from randomized 3 fields per section and 3 heart sections per mouse. Each group contains 6 mice.

Hematoxylin and Eosin (HE) staining

The hearts of the mice were perfused with cold PBS and excised. Then the hearts were fixed in 4% paraformaldehyde for 48 h and embedded in paraffin. HE staining was performed according to the manufacturer's protocols of the H&E buffer (Servicebio, G1005).

Immunofluorescence staining

For immunofluorescence, myocardium sections, CFs were fixed in 4% paraformaldehyde and permeabilized with 0.3% Triton-X100 and blocked with 3% BSA in PBS for 1 h. After that, the heart sections and cells were incubated with primary antibody against Vimentin（Abcam, ab8978）, IGFBP6 (DF8959, Affinity, USA), EGR1 (Abcam, ab300449), α-SMA (A2547, Sigma-Aldrich, USA), HA (#3724, CST, USA), Flag (F9291, Sigma-Aldrich, USA) and then incubated with Alexa Fluor® 555, 647 or 488 conjugated secondary antibodies. After rinsing with PBS, 4, 6-Diamidino-2-phenylindole (DAPI) was used for the nuclear staining. The proliferation of CFs was assessed in accordance with the manufacture's instruction by using 5-ethnyl-2-deoxyuridine (Edu) incorporation (Cat. #0078S, Beyotime Biotechnology, Beijing, China). Finally, a laser scanning confocal microscope (Olympus, Japan) was used to acquire immunofluorescence staining images and the fluorescence intensity was quantified by Image-Pro Plus software (version 6.0) and was analyzed by a person blinded to treatment.

Isolation and culture of adult mouse cardiac fibroblasts

Adult cardiac fibroblasts (CFs) were isolated as previously described with some modifications [1]. Briefly, 6-8-week-old C57BL/6J or IGFBP6$^{CF\text{-}KO}$ and IGFBP6$^{MF\text{-}KO}$ mice were executed, and their hearts were rapidly excised and washed with cold PBS. The tissues were rinsed in ice-cold Hank's

balanced salt solution (HBSS) to remove blood cells. Then we used type II collagenase (100 U/mL, Worthington, LS004176) to digest the tissues at 37°C for 20 min. The first digestion was removed, and the second digestion medium was collected in Dulbecco's modified Eagle's medium (DMEM, Gibco, USA) supplied with penicillin, streptomycin and 10% fetal bovine serum (FBS, Gibco). The procedure was repeated until all tissues were digested. The digested cell suspension was gently triturated by pipetting and then stained with a 100 μm cell strainer to eliminate tissue debris. Finally, all the medium was centrifuged for 5 min at 3000 rpm and resuspended in completed medium and then plated into 10 cm plated and attached for 1 h before the first media change to remove non-adherent cells, including myocytes and endothelial cells.

Isolation of adult mouse cardiac myocytes

Adult mouse cardiomyocytes (CMs) were isolated as previously described [2]. In brief, mice were anesthetized with 1.5% isoflurane and heart was exposed. Then 7 mL EDTA buffer (130 mM NaCl, 5 mM KCl, 0.5 mM Na_2HPO_4, 10 mM HEPES, 10 mM Taurine, 10 mM D-glucose, 10 mmol/L BDM, 5 mM EDTA, pH 7.8) was injected steadily into the right ventricle after cutting the descending aorta. The heart was then excised and sequentially perfused with 10 mL EDTA buffer, 3 mL perfusion buffer (130 mM NaCl, 5 mM KCl, 0.5 mM Na_2HPO_4, 10 mM HEPES, 10 mM Taurine, 10 mM D-glucose, 10 mmol/L BDM, 1 mM $MgCl_2$, pH 7.8) and 30 mL pre-warmed collagenase buffer (perfusion buffer with 0.5 mg/mL collagenase II, 0.5 mg/mL collagenase IV and 0.05 mg/mL protease XIV). Cardiac ventricles were further dissociated into pieces in collagenase buffer. Then we used 5 mL of stop buffer (comprising perfusion buffer with 5% FBS) to halt the digestion. The resulting cell suspension was filtered through a 100 μm filter into a 50 mL tube, allowing the formation of a pellet through gravity settling. The pellet with CMs underwent 3 steps of calcium reintroduction and were collected to analyze the expression of IGFBP6.

Collagen gel contraction assay

The ability of fibroblasts to contract collagen was assessed using the cell contraction assay (Corning, 354236, USA) per the manufacturer's protocol. The contraction assays utilized a final collagen concentration of 3.0 mg/mL and a cell concentration of 1×10^5 cells/ml within the matrix. 100% glacial acetic acid was diluted to 0.2% acetic acid solution and subsequently sterilized by filtering

it through a 0.2 μm filter and cooled to 4°C. Following sterile conditions, type I collagen was combined with the 0.2% acetic acid solution to create a 3 mg/mL collagen solution. The optimal amount of 1 M NaOH (5 μL) was added to the collagen/media mixture, using the same quantity for all subsequent gels. Each experiment was conducted at least thrice independently, and for every set of conditions, the experiment was repeated three times.

Western blotting

Myocardium tissues or cells were lysed using RIPA lysis buffer (#P0013B, Beyotime Biotechnology) containing protease inhibitor cocktail (#78438, Thermo Fisher Scientific). BCA (#23227, Thermo Fisher Scientific) method was used to quantify protein concentrations. The equal amounts of protein extracts were separated in SDS-PAGE gels and blotted into PVDF membranes. After blocked for 1.5 h in 5% non-fat milk, the membranes were incubated overnight at 4°C with primary antibodies, followed by the incubation of horseradish peroxidase-conjugated secondary antibodies for 2 h at room temperature. The signals were detected using the Electrochemiluminescence (ECL) substrate (Thermo, #32132) and images were obtained by ChemiDoc™ Imaging System (Bio-Rad, CA, US).

Co-immunoprecipitation Mass spectrometry analysis

Mass spectrometry analysis was performed as described previously [3]. IGFBP6 was immunoprecipitated using anti-IGFBP6 antibody to identify proteins interacting with IGFBP6 in cardiac fibroblasts. The gel was cut into small pieces and in-gel digestion was performed. The protein ample was denatured by 2% SDS buffer containing 50 mM DTT for 20 min RT and then boiled at 100°C for 5 min. The protein sample was alkylated for 1 hour at room temperature in the dark by addition of a final concentration of 200 mM iodoacetamide (IAA). The eluted peptides were lyophilized using a SpeedVac (ThermoSavant) and resuspended in 1% formic acid 5% acetonitrile. The peptides were re-dissolved in solvent A (A: 0.1% formic acid in water) and analyzed by Orbitrap Exploris 480 with a FAIMS coupled to an EASY-nanoLC 1200 system (Thermo Fisher Scientific, MA, USA). Raw Data of DIA were processed and analyzed by Spectronaut 14 (Biognosys AG, Switzerland).

Chromatin Immunoprecipitation (ChIP) Assays

ChIP-qPCR was performed on mouse cardiac fibroblasts using CHIP Assay Kit (#P2078, Beyotime Biotechnology). Briefly, chromatin in CFs was harvested and cross-linked with 1% formaldehyde for 10 min at room temperature and stopped by freshly prepared 0.125 mol/L Glycine. Cross-linked chromatin complexes were subsequently lysed using Nuclei Isolation Buffer (20 mM HEPES pH 7.5, 10 mM KCl, 1 mM EDTA, 0.2% NP40, 10% Glycerol, 1× Protease Inhibitor Cocktail (PIC; Roche)) and then sonicated in Sonication Buffer (20 mM Tris HCl pH 8.0, 2 mM EDTA, 150 mM NaCl, 1% NP40, 0.1% SDS, 1× PIC) using a Qsonica Q125 (80% amplitude, pulse for 20 s on and 10 s off for a total sonication "on" time of 15 min of elapsed time; Qsonica, Q125, America). Chromatin was sheared into 200-300 bp DNA fragments by ultrasound with Biorupter. After centrifugation (12,000 × g, 10 min at 4°C), the chromatin was incubated overnight with anti-EGR1 or IgG antibodies at 4°C. Then, the mixture was incubated with protein A/G agarose beads and rotated at 4°C for 1 h. The DNA-protein complexes were then eluted from the beads with elution buffer at room temperature. After reverse cross-linking and proteinase K digestion, the complexes were purified by DNA purification kit. Finally, qPCR was used to detect these purified DNA fragments and the primers of MFAP4 were used for qPCR assay.

The following primers were used for ChIP-assay of targeting genes:

Mice MFAP4, forward: 5'- AGGCCAGAGCTGGTTCTAATC-3'

Mice MFAP4, reverse: 5'- TTAGAATTAAGCGTTGGCAGC-3.

Supplementary Reference

1. Chen X, Zhang F, Hu G, Li X, Wang L, Li C, Huo C, Xu R, Hou L, Wang N *et al*: **LRRC8A critically regulates myofibroblast phenotypes and fibrotic remodeling following myocardial infarction**. *Theranostics* 2022, **12**(13):5824-5835.
2. Ackers-Johnson M, Li PY, Holmes AP, O'Brien SM, Pavlovic D, Foo RS: **A Simplified, Langendorff-Free Method for Concomitant Isolation of Viable Cardiac Myocytes and Nonmyocytes From the Adult Mouse Heart**. *Circ Res* 2016, **119**(8):909-920.
3. Song T, Zhao S, Luo S, Chen C, Liu X, Wu X, Sun Z, Cao J, Wang Z, Wang Y *et al*: **SLC44A2 regulates vascular smooth muscle cell phenotypic switching and aortic aneurysm**. *J Clin Invest* 2024, **134**(16).

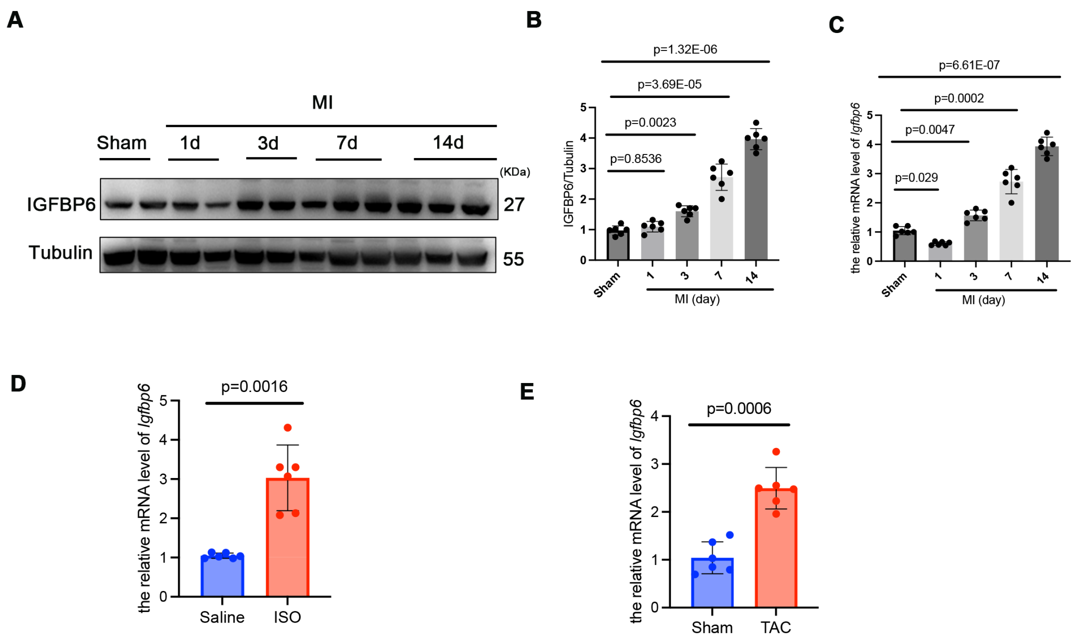

Figure S1. IGFBP6 is identified to be upregulated in the fibrotic hearts.

A-B: Wild-type (WT) mice were subjected to sham or myocardial infarction (MI) surgery at 8 weeks of age. Western blotting and quantification of IGFBP6 were performed on the heart lysates from the infarct zones at indicated time points. **C:** Real-time quantification PCR was performed to detect the expression of IGFBP6 in the heart lysates at indicated time points. n=6 mice per group. **D:** Real-time quantification PCR was performed to detect the expression of IGFBP6 in the heart lysates with saline or ISO (10 mg/kg/d, 14 days). n=6 mice per group. **E:** Real-time quantification PCR was performed to detect the expression of IGFBP6 in the TAC-induced fibrotic hearts. n=6 mice per group. Data are expressed as mean ± SD. One-way ANOVA followed by Boferroni post hoc test was used for analysis. Two tailed Student's *t*-test was used for D-E.

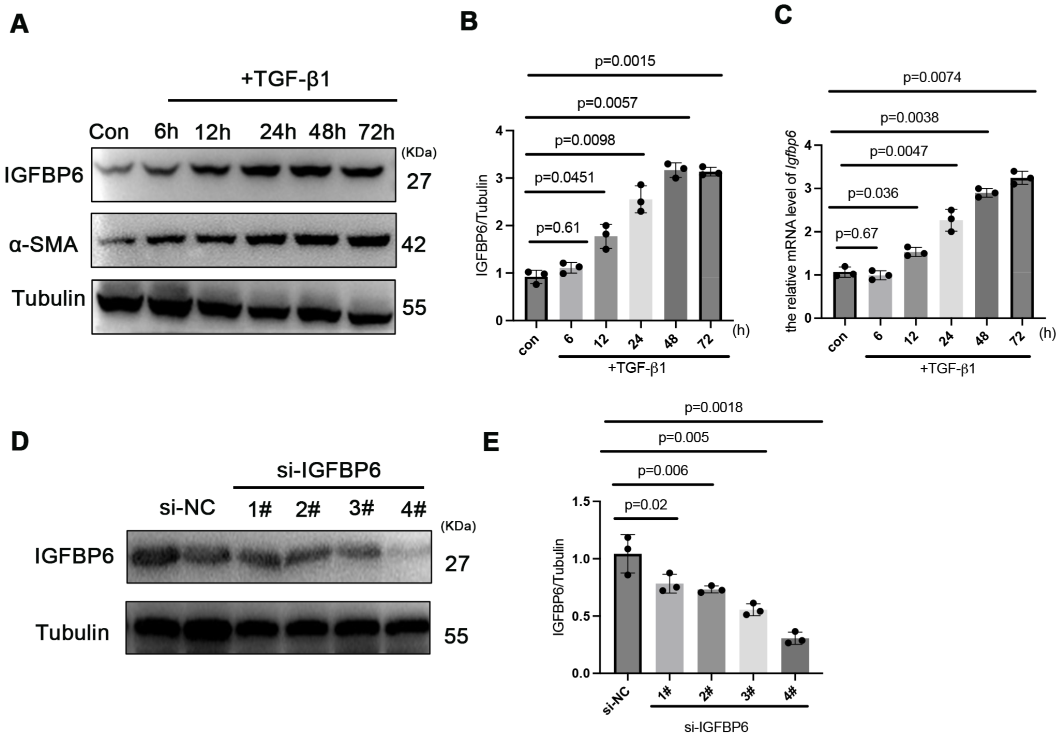

Figure S2. IGFBP6 expression is increased in CFs with the TGF-β1 challenge.

A-B: Cardiac fibroblasts were isolated and cultured and then treated with TGF-β1(10 ng/mL) for different time points. Western blotting analysis and quantification of IGFBP6 was shown. n=3 independent biological. **C:** Real-time quantification PCR was performed to detect the expression of IGFBP6 in the CFs with the TGF-β1 stimulation at indicated time points. n=3 independent biological. **D-E:** Primary cardiac fibroblasts were isolated from the 6-week-old male C57BL/6J mice. Fibroblasts were transfected with small interfering RNA targeting IGFBP6 (si-IGFBP6) or negative control (si-NC). Western blotting and quantification of silencing IGFBP6. n=3 independent biological. Data are expressed as mean ± SD. One-way ANOVA followed by Boferroni post hoc test was used for analysis.

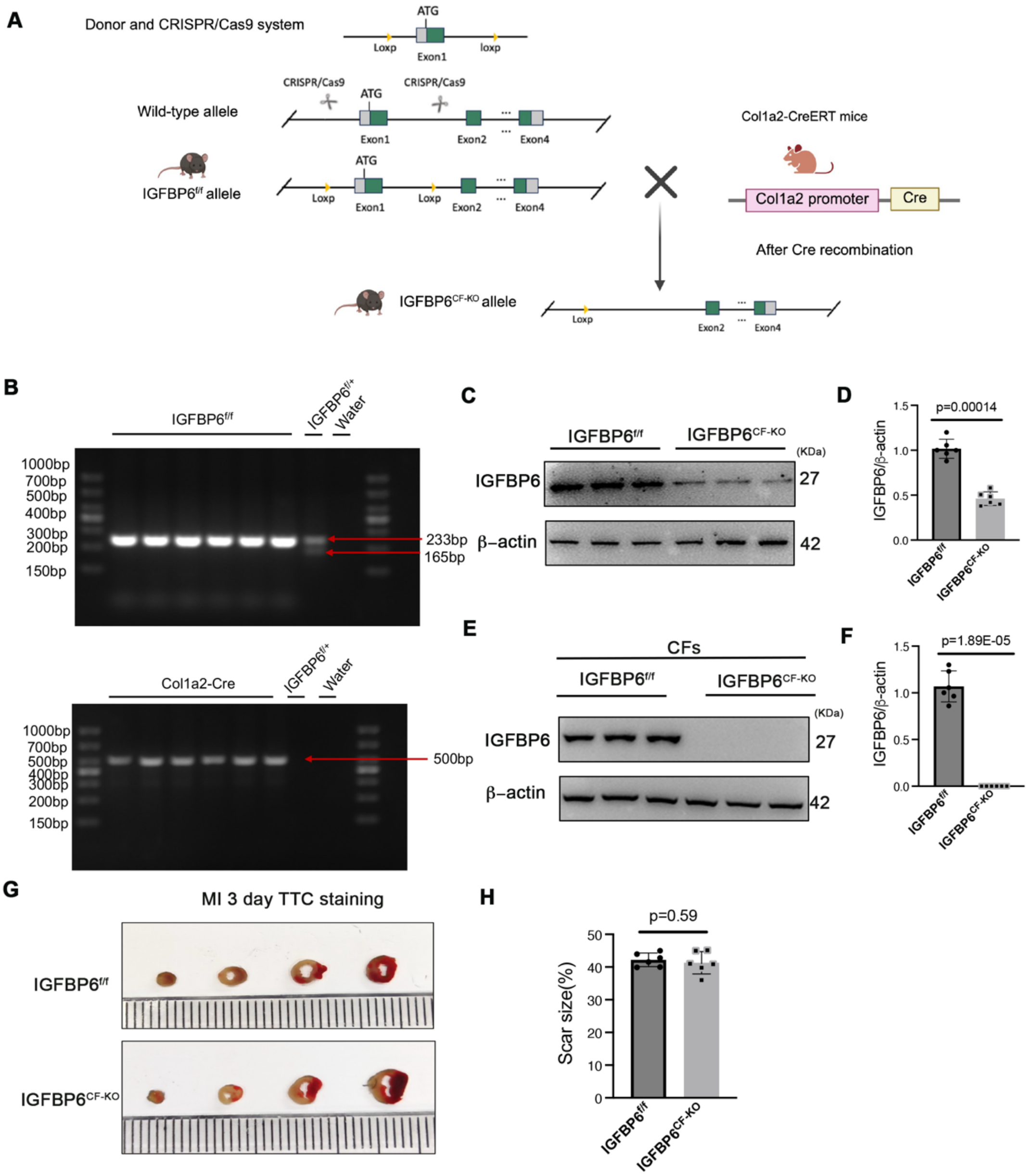

A
Donor and CRISPR/Cas9 system
ATG
Loxp
loxp
Exon1
Wild-type allele
CRISPR/Cas9
Exon2
Exon4
IGFBP6f/f allele
Col1a2-CreERT mice
Col1a2 promoter
Cre
After Cre recombination
IGFBP6CF-KO allele
B
IGFBP6f/f
IGFBP6f/+
Water
1000bp
700bp
500bp
400bp
300bp
200bp
150bp
233bp
165bp
Col1a2-Cre
500bp
C
IGFBP6f/f
IGFBP6CF-KO
(KDa)
IGFBP6
27
β-actin
42
D
p=0.00014
IGFBP6/β-actin
E
CFs
F
p=1.89E-05
G
MI 3 day TTC staining
H
p=0.59
Scar size(%)

Figure S3. Construction of cardiac fibroblast-specific IGFBP6 knockout mice.

A: Schematic diagram for generation of cardiac fibroblasts (CFs) specific IGFBP6 deficiency mouse line. IGFBP6 conditional knockout by crossing IGFBP6$^{f/f}$ mice with CFs specific Col1a2-CreERT mice. **B:** PCR analysis of DNA isolated from tails in IGFBP6$^{f/+}$, IGFBP6$^{f/f}$ and Col1a2+ mice. The PCR primers used for genotype identification of IGFBP6$^{f/f}$ and Col1a2-CreERT transgenic mice. **C-D:** The expression of IGFBP6 was examined by western blot following 5 continuous days injection of tamoxifen in IGFBP6$^{f/f}$ and IGFBP6$^{CF\text{-}KO}$ mice myocardium. n=6 mice per group. **E-F:** The expression of IGFBP6 was measured in cardiac fibroblasts isolated from IGFBP6$^{f/f}$ and IGFBP6$^{CF\text{-}KO}$ mice. n=6 mice per group. **G-H:** IGFBP6$^{f/f}$ and IGFBP6$^{CF\text{-}KO}$ mice were subjected to MI operation and the infarct size were evaluated by triphenyl tetrazolium chloride (TTC) staining at 3 days post-MI. n=6 mice per group. Data are expressed as mean ± SD. Data were analyzed by unpaired student's t test.

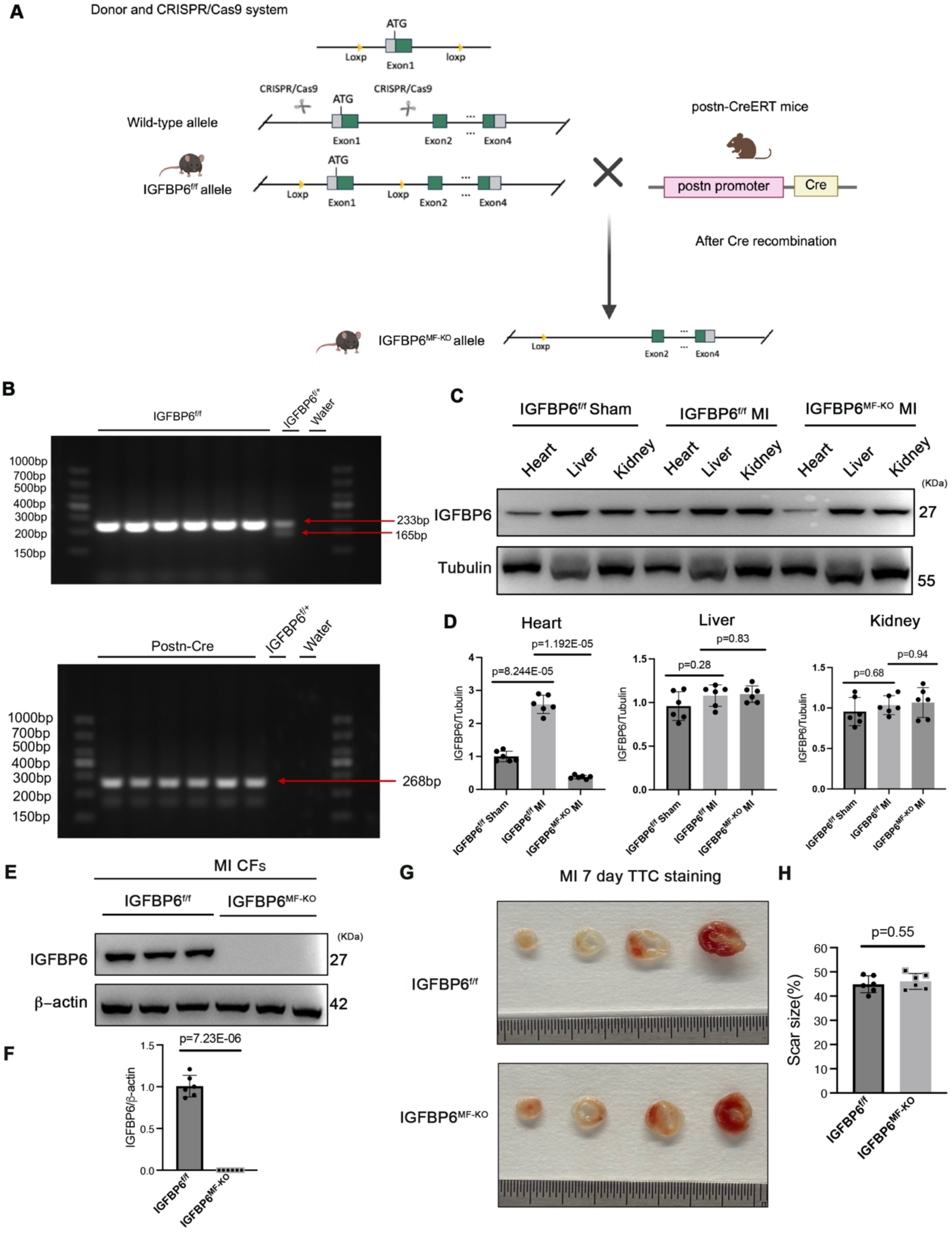
A
Donor and CRISPR/Cas9 system
ATG
Loxp
Exon1
loxp
CRISPR/Cas9
CRISPR/Cas9
Wild-type allele
Exon2
Exon4
postn-CreERT mice
IGFBP6f/f allele
Loxp
postn promoter
Cre
After Cre recombination
IGFBP6MF-KO allele
B
IGFBP6f/f
IGFBP6f/+
Water
1000bp
700bp
500bp
400bp
300bp
200bp
150bp
233bp
165bp
Postn-Cre
268bp
C
IGFBP6f/f Sham
IGFBP6f/f MI
IGFBP6MF-KO MI
Heart
Liver
Kidney
(KDa)
IGFBP6
27
Tubulin
55
D
IGFBP6/Tubulin
p=8.244E-05
p=1.192E-05
p=0.28
p=0.83
p=0.68
p=0.94
IGFBP6f/f Sham
IGFBP6f/f MI
IGFBP6MF-KO MI
E
MI CFs
IGFBP6f/f
IGFBP6MF-KO
β-actin
42
F
IGFBP6/β-actin
p=7.23E-06
G
MI 7 day TTC staining
H
Scar size(%)
p=0.55

Figure S4. Construction of myofibroblast-specific IGFBP6 knockout mice.

A: Schematic diagram for generation of myofibroblasts (MFs) specific IGFBP6 deficiency mouse line. IGFBP6 conditional knockout by crossing IGFBP6$^{f/f}$ mice with MFs specific Postn-CreERT mice. **B:** PCR analysis of DNA isolated from tails in IGFBP6$^{f/+}$, IGFBP6$^{f/f}$ and Postn-Cre mice. **C-D:** The expression of IGFBP6 was examined by western blot following 5 continuous days injection of tamoxifen in IGFBP6$^{f/f}$ and IGFBP6$^{MF\text{-}KO}$ mice myocardium at 7 days after MI. n=6 mice per group. **E-F:** The expression of IGFBP6 was measured in fibroblasts isolated from IGFBP6$^{f/f}$ and IGFBP6$^{MF\text{-}KO}$ mice post-MI. n=6 mice per group. **G-H:** IGFBP6$^{f/f}$ and IGFBP6$^{MF\text{-}KO}$ mice were subjected to MI operation and the infarct size were evaluated by triphenyl tetrazolium chloride (TTC) staining at 7 days post-MI. n=6 mice per group. Data are expressed as mean ± SD. Data were analyzed by unpaired student's t test. One-way ANOVA followed by Boferroni post hoc test was used for analysis.

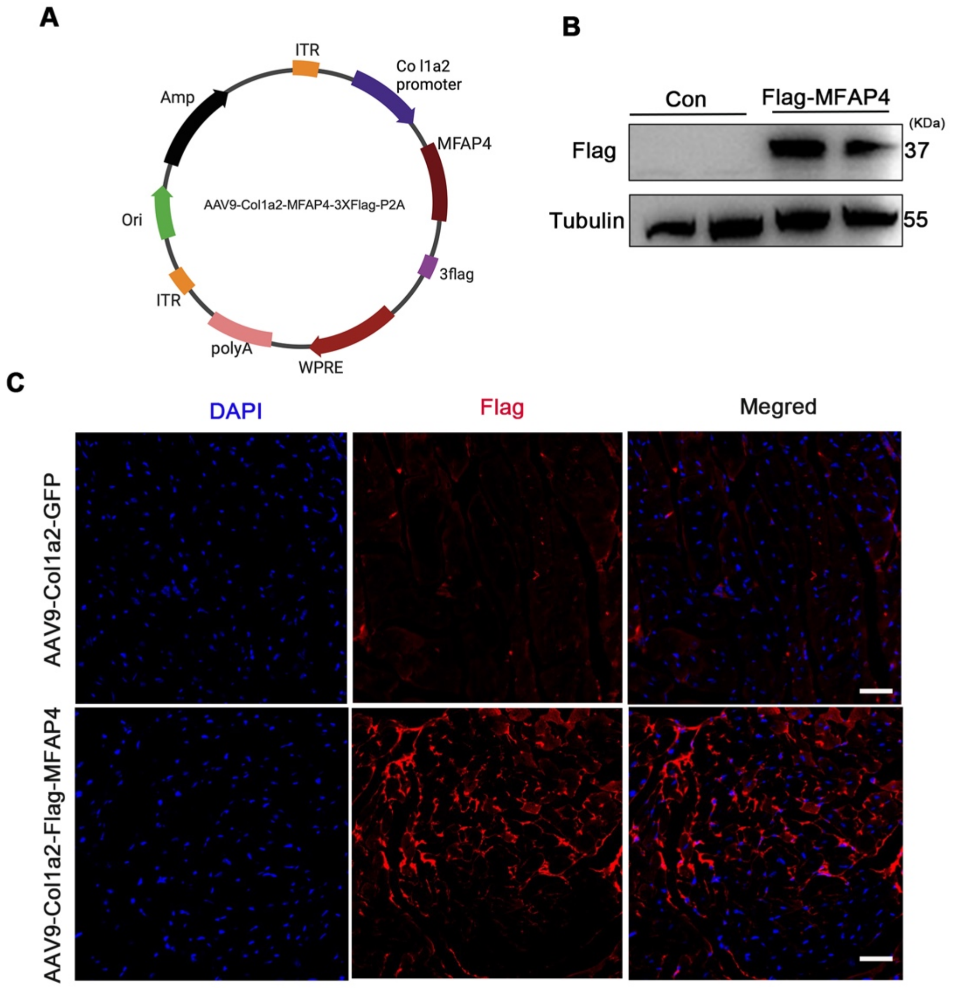

Figure S5. Construction of cardiac fibroblasts-specific MFAP4 overexpression mice.

A: Schematic representation of rAAV9-Col1a2-MFAP4-3×Flag vector construct. **B:** Verification of overexpression of MFAP4 by immunoblot analysis of cell lysates from hearts of indicated mice. **C:** Immunofluorescence examination of Flag (red) infected with rAAV9-Col1a2-MFAP4-3×Flag mice. Scale bars: 20 μm.

Supplemental Tables

Table S1. Clinical Characteristics of patients.

Sample	Healthy	MI
Characteristics	24	32
Gender(male)	16	24
Age, years	59.3±4.2	60.19±3.2
BMI	23.32±2.07	24.39±2.25
BNP, pg/mL	45±22.21	712±102.2
CTnI, pg/mL	0.0133±0.024	0.762±0.564
CK, U/L	42±34	102±0.32
CK-MB, U/L	4.76±3.46	14.79±2.48
Glucose, mmol/L	5.34±1.1	5.537±1.26
TC, mmol/L	4.10±0.32	4.06±0.68
TG, mmol/L	0.6±0.17	0.75±0.12
ALT, U/L	23.55±4.67	32.68±3.56
AST, U/L	22.17±3.229	26.87±4.698
BUN, mmol/L	4.1±0.5	4.35±1.2
CREA, mmol/L	62.7±6.9	69.6±13.6
CRP, mg/mL	2.15±1.229	26.43±4.18

BMI: Body Mass Index; BNP: Brain Natriuretic Peptide; CTnI: Cardiac Troponin I; CK: Creatine Kinase; CK-MB: Creatine Kinase Isoenzymes; TC: Total Cholesterol; TG: Triglyceride; ALT: Alanine Aminotransferase; AST: Aspartate Aminotransferase; BUN: Blood Urea Nitrogen; CREA: Creatinine; CRP: C-reactive Protein.

Table S2. Primer sequences for qRT-PCR.

Gene	Forward (5'-3')	Reverse (5'-3')
Mus *α-SMA*	CTTCGTGACTACTGCCGAGC	AGGTGGTTTCGTGGATGCC
Mus *Col1α1*	GCTCCTCTTAGGGGCCACT	ATTGGGGACCCTTAGGCCAT
Mus *Col3α1*	CCCTGGACCTCAGGGTATCA	GGGTTTCCATCCCTTCCAGG
Mus *Ctgf*	CTGCGAGGAGTGGGTGTG	ATGTGTCTTCCAGTCGGTAGG
Mus *Igfbp6*	AAGTCTCAAGGTTATAGGGACGG	CCATGCTTGTCTGGGTATAGTGT
Mus *Mfap4*	GCAACCCCTGGACTGTGATG	TTGTCATGTCGCAGAAGACGG
Mus *Hprt*	GTTGGATACAGGCCAGACTTTGTT	GATTCAACTTGCGCTCATCTTAGGC

Table S3. Cardiac function of IGFBP6$^{f/f}$, IGFBP6^{CF-KO} and IGFBP6^{MF-KO} mice at baseline.

Group	IGFBP6$^{f/f}$	IGFBP6^{CF-KO}	IGFBP6^{MF-KO}
N	10	10	10
HR（bpm)	460±58	456±40	446±52
LVIDd; mm	2.13±0.42	2.19±0.32	2.15±0.36
LVIDs; mm	2.76±0.46	2.79±0.48	2.7±0.56
LVESV	17.22±3.65	17.37±3.26	17.37±3.26
LVEDV	49.10±4.32	49.16±4.68	49.16±4.68
%EF	66.50±4.67	66.75±4.62	66.75±4.62
%FS	36.44±4.12	37.76±4.23	36.76±3.24

Data are expressed as mean ± SD and compared using unpaired student’s t test followed by Bonferroni’s multiple comparisons test. There is no significant difference of cardiac function between IGFBP6$^{f/f}$, IGFBP6^{CF-KO}, IGFBP6^{MF-KO} mice at baseline.

Table S4. Cardiac fibroblasts-specific IGFBP6 knockout improves cardiac function post-MI.

	IGFBP6$^{f/f}$ Sham	IGFBP6$^{f/f}$ MI	IGFBP6$^{CF\text{-}KO}$ Sham	IGFBP6$^{CF\text{-}KO}$ MI
N	8	8	8	8
HR（bpm)	450±28	454±37	436±40	456±42
LVIDd; mm	2.23±0.43	4.57±0.48**	2.29±0.32	3.60±0.22$^{\#}$
LVIDs; mm	2.78±0.56	3.43±0.27**	2.89±0.28	2.85±0.39$^{\#}$
LVESV	17.12±2.65	31.60 ±4.43***	15.37±4.26	25.47±4.10$^{\#\#}$
LVEDV	49.50±5.32	68.56±5.28**	49.16±4.87	59.01±3.21$^{\#}$
%EF	70.12±6.842	40.84±4.28***	64.75±7.62	55.63±4.236$^{\#\#}$
%FS	40.97±2.411	19.19±1.219**	39.91±4.03	25.99±2.62$^{\#\#}$

Data are expressed as mean ± SD and compared using ordinary two-way ANOVA followed by Bonferroni's multiple comparisons test.

**$p < 0.01$, ***$p < 0.001$ compared to IGFBP6$^{f/f}$ Sham group.

$^{\#}$ $p < 0.05$, $^{\#\#}$ $p < 0.01$ compared to IGFBP6$^{f/f}$ MI group.

Table S5. Cardiac fibroblasts-specific IGFBP6 knockout improves cardiac function with ISO-challenge.

	IGFBP6$^{f/f}$ Saline	IGFBP6$^{f/f}$ ISO	IGFBP6$^{CF\text{-}KO}$ Saline	IGFBP6$^{CF\text{-}KO}$ ISO
N	8	8	8	8
HR（bpm)	440±30	504±42	448±30	496±48
LVIDd; mm	2.23±0.43	4.95±0.28**	2.29±0.32	3.72±0.22$^{\#}$
LVIDs; mm	2.62±0.50	3.76±0.47**	2.76±0.32	2.82±0.32$^{\#}$
LVESV	18.12±2.46	30.40 ±4.20***	15.20±4.26	25.47±4.10$^{\#\#}$
LVEDV	48.60±5.40	66.58±5.60**	48.60±4.60	58.60±3.12$^{\#}$
%EF	68.87±3.937	41.09±3.708***	66.91±4.533	53.2±3.955$^{\#\#}$
%FS	41.96±4.64	20.19±2.08**	42.54±2.99	27.92±2.251$^{\#\#}$

Data are expressed as mean ± SD and compared using ordinary two-way ANOVA followed by Bonferroni's multiple comparisons test.

** $p < 0.01$, *** $p < 0.001$ compared to IGFBP6$^{f/f}$ Saline group.

$^{\#}$ $p < 0.05$, $^{\#\#}$ $p < 0.01$ compared to IGFBP6$^{f/f}$ ISO group.

Table S6. IGFBP6 deficiency in myofibroblasts attenuated MI Induced cardiac dysfunction.

	IGFBP6$^{f/f}$ Sham	IGFBP6$^{f/f}$ MI	IGFBP6$^{MF\text{-}KO}$ Sham	IGFBP6$^{MF\text{-}KO}$ MI
N	8	8	8	8
HR（bpm)	450±28	454±37	436±40	456±42
LVIDd; mm	2.23±0.43	4.57±0.48**	2.29±0.32	3.60±0.22$^{\#}$
LVIDs; mm	2.78±0.56	3.43±0.27**	2.89±0.28	2.85±0.39$^{\#}$
LVESV	17.12±2.65	31.60 ±4.43***	15.37±4.26	25.47±4.10$^{\#\#}$
LVEDV	49.50±5.32	68.56±5.28**	49.16±4.87	59.01±3.21$^{\#}$
%EF	67.29±3.387	39.59±4.638***	66.91±4.533	49.95±3.534$^{\#\#\#}$
%FS	41.09±2.404	19.41±2.688**	39.52±3.636	27.32±2.812$^{\#\#}$

Data are expressed as mean ± SD and compared using ordinary two-way ANOVA followed by Bonferroni’s multiple comparisons test.

** $p < 0.01$, *** $p < 0.001$ compared to IGFBP6$^{f/f}$ Sham group.

$^{\#}$ $p < 0.05$, $^{\#\#}$ $p < 0.01$, $^{\#\#\#}$ $p < 0.001$ compared to IGFBP6$^{f/f}$ MI group.

Table S7. IGFBP6 deficiency in myofibroblasts attenuated ISO-Induced cardiac dysfunction.

	**IGFBP6$^{f/f}$ Saline**	**IGFBP6$^{f/f}$ ISO**	**IGFBP6$^{MF\text{-}KO}$ Saline**	**IGFBP6$^{MF\text{-}KO}$ ISO**
N	8	8	8	8
HR（bpm)	420±38	492±30	428±38	476±28
LVIDd; mm	2.23±0.43	4.95±0.28**	2.29±0.32	5.60±0.22$^{\#}$
LVIDs; mm	2.62±0.50	3.76±0.47**	2.76±0.32	4.42±0.32$^{\#}$
LVESV	18.12±2.46	30.40 ±4.20***	15.20±4.26	25.47±4.10$^{\#\#}$
LVEDV	48.60±5.40	66.58±5.60**	48.60±4.60	58.60±3.12$^{\#}$
%EF	66.91±4.533	39.84±3.905***	70.79±3.793	52.88±3.982$^{\#\#}$
%FS	39.52±2.634	19.31±2.223**	43.96±2.511	26.69±2.77$^{\#\#}$

Data are expressed as mean ± SD and compared using ordinary two-way ANOVA followed by Bonferroni's multiple comparisons test.

** $p < 0.01$, *** $p < 0.001$ compared to IGFBP6$^{f/f}$ Saline group.

$^{\#}$ $p < 0.05$, $^{\#\#}$ $p < 0.01$ compared to IGFBP6$^{f/f}$ ISO group.

Table S8. MFAP4 is essential for the regulatory function of IGFBP6 in cardiac ischemic injury post-MI

	$IGFBP6^{f/f}$ Sham	$IGFBP6^{f/f}$ MI	Flag-MFAP4 MI	$IGFBP6^{CF-KO}$ MI	$IGFBP6^{CF-KO}$ + MFAP4-MI
N	8	8	8	8	8
HR（bpm)	460±38	484±42	468±38	476±38	496±28
LVIDd; mm	2.23±0.43	4.95±0.28**	5.29±0.32	$3.60±0.22^{\#}$	$3.72±0.24^{\$}$
LVIDs; mm	2.62±0.50	3.76±0.47**	4.26±0.32	$3.22±0.32^{\#}$	$3.62±0.32^{\$}$
LVESV	18.12±2.46	30.40 ±4.20***	15.20±4.26	$25.47±4.10^{\#\#}$	$25.47±4.10^{\$\$}$
LVEDV	48.60±5.40	66.58±5.60**	48.60±4.60	$58.60±3.12^{\#}$	$58.60±3.12^{\$}$
%EF	68.9±3.91	37.9±2.74***	30.2±2.44	$47.3±3.64^{\#\#}$	$40.1±4.99^{\$\$}$
%FS	38.7±2.05	18.8±1.77**	15.24±1.768	$23.4±2.83^{\#\#}$	$18.1±2.35^{\$\$}$

Data are expressed as mean ± SD and compared using ordinary two-way ANOVA followed by Bonferroni’s multiple comparisons test.

** $p < 0.01$, *** $p < 0.001$ compared to $IGFBP6^{f/f}$ Sham group.

$^{\#}$ $p < 0.05$, $^{\#\#}$ $p < 0.01$ compared to $IGFBP6^{f/f}$ MI group

$^{\$}$ $p < 0.05$, $^{\$\$}$ $p < 0.01$ compared to $IGFBP6^{CF-KO}$ MI group
